# ZW sex-chromosome evolution and contagious parthenogenesis in Artemia brine shrimp

**DOI:** 10.1093/genetics/iyac123

**Published:** 2022-08-17

**Authors:** Marwan Elkrewi, Uladzislava Khauratovich, Melissa A Toups, Vincent Kiplangat Bett, Andrea Mrnjavac, Ariana Macon, Christelle Fraisse, Luca Sax, Ann Kathrin Huylmans, Francisco Hontoria, Beatriz Vicoso

**Affiliations:** Institute of Science and Technology Austria, Klosterneuburg 3400, Austria; Institute of Science and Technology Austria, Klosterneuburg 3400, Austria; Department of Chromosome Biology, Max Perutz Labs, University of Vienna, Vienna 1030, Austria; Institute of Science and Technology Austria, Klosterneuburg 3400, Austria; Faculty of Science and Technology, Department of Life and Environmental Sciences, Bournemouth University, Poole BH12 5BB, UK; Institute of Science and Technology Austria, Klosterneuburg 3400, Austria; Institute of Science and Technology Austria, Klosterneuburg 3400, Austria; Institute of Science and Technology Austria, Klosterneuburg 3400, Austria; Institute of Science and Technology Austria, Klosterneuburg 3400, Austria; CNRS, Univ. Lille, UMR 8198—Evo-Eco-Paleo, 59000 Lille, France; Institute of Science and Technology Austria, Klosterneuburg 3400, Austria; Lewis and Clark College, Portland, OR 97219, USA; Institute of Science and Technology Austria, Klosterneuburg 3400, Austria; Institute of Organismic and Molecular Evolution, Johannes Gutenberg Universität Mainz, Mainz 55122, Germany; Instituto de Acuicultura de Torre de la Sal (IATS-CSIC), 12595 Ribera de Cabanes (Castellón), Spain; Institute of Science and Technology Austria, Klosterneuburg 3400, Austria

**Keywords:** sex chromosome, female heterogamety, asexuality, dosage compensation

## Abstract

Eurasian brine shrimp (genus *Artemia*) have closely related sexual and asexual lineages of parthenogenetic females, which produce rare males at low frequencies. Although they are known to have ZW chromosomes, these are not well characterized, and it is unclear whether they are shared across the clade. Furthermore, the underlying genetic architecture of the transmission of asexuality, which can occur when rare males mate with closely related sexual females, is not well understood. We produced a chromosome-level assembly for the sexual Eurasian species *Artemia sinica* and characterized in detail the pair of sex chromosomes of this species. We combined this new assembly with short-read genomic data for the sexual species *Artemia* sp. *Kazakhstan* and several asexual lineages of *Artemia parthenogenetica*, allowing us to perform an in-depth characterization of sex-chromosome evolution across the genus. We identified a small differentiated region of the ZW pair that is shared by all sexual and asexual lineages, supporting the shared ancestry of the sex chromosomes. We also inferred that recombination suppression has spread to larger sections of the chromosome independently in the American and Eurasian lineages. Finally, we took advantage of a rare male, which we backcrossed to sexual females, to explore the genetic basis of asexuality. Our results suggest that parthenogenesis is likely partly controlled by a locus on the Z chromosome, highlighting the interplay between sex determination and asexuality.

## Introduction

The diversity of reproductive and sex-determining systems has long puzzled evolutionary biologists (Bachtrog *et al.* 2014; Pennell *et al.* 2018; Picard *et al.* 2021). When separate sexes are present, the development of males and females can be controlled by environmental factors or through the presence of sex-determining loci (Beukeboom and Perrin 2014; Bachtrog *et al.* 2014). These sex determining loci are typically carried by specialized “sex chromosomes,” such as the X and Y chromosomes of mammals. Sex chromosomes initially arise from standard pairs of autosomes, but can progressively stop recombining over much of their length, ultimately resulting in genetic and morphological differentiation (Charlesworth *et al.* 2005; Wright *et al.* 2016). Each segment of the sex chromosome pair that stopped recombining at a given timepoint is referred to as a “stratum,” and strata of different ages are often found on the same pair of sex chromosomes (Lahn and Page 1999; Handley *et al.* 2004). The Y chromosome stops recombining altogether after XY recombination suppression and eventually degenerates, i.e. it accumulates deleterious mutations and can lose many or even all of its genes (Bachtrog 2013). This gene loss leads to dosage deficits in males, since many X-linked genes become single-copy. Mechanisms of dosage compensation often target the X-chromosome and regulate its expression, thereby reestablishing optimal dosage balance of genes across the genome (Charlesworth 1978; Straub and Becker 2007; Vicoso and Bachtrog 2009; Disteche 2016). Alternatively, both the silencing of Y-linked genes and compensation of X-linked genes may arise concurrently as a result of runaway regulatory divergence that sets up and reinforces the predominance of X over Y expression (Lenormand *et al.* 2020; Lenormand and Roze 2022). Much of our understanding of these processes has come from studying the ancient XY systems of traditional model organisms such as mice and fruit flies. Despite the recent characterization of young sex chromosomes in many nonmodel species (Charlesworth 2019), many questions remain about the earlier stages of sex-chromosome divergence. For example, what molecular mechanisms and selective pressures drive the initial loss of recombination between sex chromosomes (Ponnikas *et al.* 2018)? Similarly, female-heterogametic species (i.e. females are ZW, males are ZZ) have remained relatively understudied, as they are not found in any of the main model organisms. While parallels exist between the evolution of XY and ZW pairs, such as the progressive loss of recombination and subsequent degradation of the Y/W-chromosomes (Ellegren 2011; Vicoso *et al.* 2013; Zhou *et al.* 2014; Picard *et al.* 2018; Sigeman *et al.* 2021), some aspects of their evolution seem to differ. In particular, dosage compensation of Z-chromosomes is often limited to a few dosage-sensitive genes [i.e. it works gene-by-gene, as opposed to the chromosome-wide mechanisms found in many XY species (Mank 2013; Rovatsos and Kratochvíl 2021)]. These discrepancies may have to do with systematic differences in selection and mutation between males and females (Vicoso and Bachtrog 2009; Ellegren 2011; Mullon *et al.* 2015), or may simply be a coincidence due to the few ZW systems characterized in detail at the molecular level (Rovatsos and Kratochvíl 2021).

Although the prevalence of sexual reproduction suggests that it offers long-term advantages, asexual lineages are found in many clades and successfully inhabit a variety of ecological niches (Toman and Flegr 2018). Transitions from sexual to asexual reproduction are frequent (Neiman *et al.* 2014), and can involve a diversity of mechanisms that disrupt meiosis, such as novel mutations, hybridization of closely related lineages, and polyploidization (Neiman *et al.* 2014). Asexuality can evolve from any ancestral sex-determining system, including in species with differentiated sex chromosomes (e.g. Schwander and Crespi 2009; Jaquiéry *et al.* 2014; Mignerot *et al.* 2019), and understanding the mechanisms underlying these transitions has been a key goal of the field.

In many asexual lineages, males are occasionally produced, and can fertilize closely related sexual females, which then give rise to new asexual lineages (“contagious parthenogenesis”). These crosses have facilitated the use of traditional genetic approaches for understanding the genetic architecture of asexuality (Jaquiéry *et al.* 2014). Transitions from sexual to asexual reproduction have primarily been studied in animal species where both sexual reproduction and parthenogenesis were ancestrally part of the life cycle, either in the form of cyclical parthenogenesis or haplodiploidy (Neiman *et al.* 2014). In this case, the loss of sexual reproduction and consequent obligatory parthenogenesis is often controlled by 1 or only a few loci (Lynch *et al.* 2008; Sandrock and Vorburger 2011; Eads *et al.* 2012; Jaquiéry *et al.* 2014; Aumer *et al.* 2017; Yagound *et al.* 2020). In the pea aphid, the locus controlling asexuality is found on the X-chromosome (Jaquiéry *et al.* 2014), and a locus of large effect on parthenogenesis was also found on the UV sex chromosome pair of brown algae *Ectocarpus* (Mignerot *et al.* 2019), raising interesting questions about the interplay between the ancestral sex-determining system and contagious parthenogenesis. One direct link between the 2 phenomena is that when asexuals are derived from an ancestral XX/XY or haplodiploid sex-determination systems, rare males can be formed through the loss of an X-chromosome (Kampfraath *et al.* 2020) or through accidental production of haploid individuals during automixis (Sandrock and Vorburger 2011). Less is known about the creation of rare males when the ancestral sex-determination system was female-heterogamety. More generally, it is unclear if sex chromosomes are a prime spot for the location of genes regulating asexual reproduction, since very few transitions have been characterized in organisms with sex chromosomes.

Brine shrimp of the genus *Artemia* have both asexual and sexual species (Abatzopoulos 2018), as well as ZW sex chromosomes with putative ancient and recent strata (Bowen 1963; De Vos *et al.* 2013; Accioly *et al.* 2014; Huylmans *et al.* 2019), making them an ideal model for addressing many of these questions. While all American species are sexual, the Eurasian clade consists of a few sexual species (including *Artemia sinica*, *Artemia* sp. *Kazakhstan*, and *Artemia urmiana*) and of various asexual lineages (collectively refered to as *Artemia parthenogenetica*, and further referred to by their location of origin) (Van Stappen 2002; Maccari, Amat, *et al.* 2013). Asexuals vary in ploidy, but only diploid lineages are considered here (Maccari, Amat, *et al.* 2013). Originally thought of as ancient, these lineages turned out to have arisen recently through hybridization between asexual lineages and individuals from, or closely related to, *A.* sp. *Kazakhstan* (Baxevanis *et al.* 2006; Maccari, Amat, *et al.* 2013; Rode *et al.* 2022). In *Artemia*, such contagious parthenogenesis can occur through the production of rare males by asexual lineages, which can fertilize closely related sexual females (Maccari, Gómez *et al.* 2013; Abatzopoulos 2018). Furthermore, asexual females can mate with males of sexual species and produce a minority of offspring sexually (Boyer *et al.* 2021). The ZW pair of *Artemia* has been mostly studied in the American species *Artemia franciscana* (Bowen 1963; Parraguez *et al.* 2009; De Vos *et al.* 2013; Accioly *et al.* 2014)*.* Both a small differentiated region and a nonrecombining but largely undifferentiated region were detected, making it an interesting system to understand the first steps leading to ZW divergence (Huylmans *et al.* 2019). Gene expression in the differentiated region appears to be fully balanced between males and females, but there was limited power to detect changes due to the fragmented nature of the genome (Huylmans *et al.* 2019). Eurasian lineages also carry a ZW pair (Haag *et al.* 2017), but whether the same chromosome is used for sex determination across the clade in not known. Because *A. parthenogenetica* reproduce through central fusion automixis (Nougué *et al.* 2015), a modified form of meiosis, which allows for loss of heterozygosity when recombination between chromosomes occurs, rare recombination events between the Z and W (which replace part of the W with its Z-linked homologous region) can lead to the creation of rare males (Nougué *et al.* 2015; Boyer *et al.* 2022). Finally, the genetic mechanisms behind asexuality, and whether the sex chromosomes play any further role in its evolution, have not yet been explored in detail.

Here, we develop several genomic resources for *Artemia* lineages, including the first chromosome-level assembly for the *Artemia* genus (*A. sinica*), as well as short-read genomic data for *A.* sp. *Kazakhstan* and several lineages of *A. parthenogenetica* (see Supplementary Fig. 1 for a phylogeny of the lineages). Using these data, we are able to provide an in-depth characterization of sex-chromosome evolution across the genus, including identifying an ancient region shared with the American species *A. franciscana*. Finally, we find evidence that asexuality is likely partly controlled by a locus on the Z chromosome—a first in a ZW sex chromosome system.

## Materials and methods

### Sampling and DNA extractions

Cysts from *A. sinica* (originally from Tanggu salterns, PR China), *A.* sp. *Kazakhstan* (originally from an unknown location in Kazakhstan), and 2 lineages of *A. parthenogenetica* [from Lake Aibi (PR China) and from Lake Urmia (Iran)] were obtained from the Instituto de Acuicultura de Torre de la Sal (C.S.I.C.) Artemia cyst collection in Spain, as described in Huylmans *et al.* (2021). Cysts were hatched under 30 g/l salinity and grown to adulthood under 60 g/l salinity. Some of these F0 individuals were used directly for DNA extractions with the Qiagen DNeasy Blood & Tissue kit. We also set up iso-female lines in *A. sinica* and *A.* sp. *Kazakhstan*, and subjected them to 6 generations of sib–sib mating to reduce the amount of heterozygosity. Male and female individuals from *A. sinica* and *A.* sp. *Kazakhstan* inbred iso-female lines were used individually for DNA extractions with Qiagen DNeasy Blood & Tissue kit. Furthermore, 20 males and 17 females of *A. sinica* (also from the inbred iso-female line) were pooled and high molecular weight DNA was extracted using the Qiagen Genomic-tip 20/G kit.

### DNA short- and long-read sequencing

PacBio libraries were prepared and sequenced at the Vienna Biocenter Sequencing facility for the male and female *A. sinica* high molecular weight DNA. All other DNA samples were used for Illumina paired-end sequencing. Libraries were prepared and sequenced at the Vienna Biocenter Sequencing Facility. Finally, 1 male was frozen and provided to the sequencing facility for Hi-C library preparation and Illumina sequencing on a NovaSeq machine. The final list of samples, as well as the parts of the analysis that they were used in, are listed in Supplementary Table 1.

### Genome assemblies

The male PacBio reads were assembled using 2 different genome assemblers: Flye (v.2.7.1, Kolmogorov *et al.* 2019) and Miniasm (0.3-r179, minimap2 2.18-r1028-dirty was used for mapping and the consensus was generated using Racon v1.4.22) (Li 2016; Vaser *et al.* 2017). The Flye assembly was polished using male *A. sinica* short genomic reads (trimmed with the Trimmomatic package, Bolger *et al.* 2014), and the Miniasm assembly was polished using the same male short reads using the wtpoa-cns tool from wtdbg2 (version 2.5, Ruan and Li 2020). The 2 assemblies were then merged using quickmerge (version 0.3, Chakraborty *et al.* 2016) with the Miniasm assembly as the query and the Flye assembly as the reference. The resulting assembly was purged based on the male pacbio read depth to remove duplicates and contig overlaps using the purge_dups program (version 1.2.5, Guan *et al.* 2020).

To scaffold the assembly into pseudo-chromosomes, PCR duplicates were first removed from the Hi-C data using the clumpify.sh script from the BBMap package (Bushnell 2014), and the Hi-C reads were then mapped to the genome assembly using the Arima mapping pipeline with MAPQ 5 (Arima Genomics 2021) and then scaffolded using the YaHS tool (pre-release of version 1.1, Zhou *et al.* 2022). The contact maps were visualized and manually edited on Juicebox (version 1.11.08, Robinson *et al.* 2018) to generate the final chromosome-level assembly.

The female *A. sinica* genome was assembled from female PacBio reads using Flye (version 2.7.1), and it was not polished to avoid collapsing the Z and the W scaffolds. To identify putative W scaffolds, short genomic reads from 2 *A. sinica* males and 2 females were mapped to the female assembly using Bowtie2 (Langmead and Salzberg 2012). We then counted how many male and female reads mapped to each scaffold, after filtering for alignments without mismatches (by selecting only mapped reads with the CIGAR string “NM:i:0”). The female-specific k-mers inferred to obtain W-specific transcripts (section *Identification of candidate W-genes with k-mer analysis* below) were similarly mapped to each scaffold with Bowtie2 and counted. Scaffolds which had more than 5 female-specific k-mers, and more perfect matches in females than in males [male/(male + female) ≤ 0.3] were considered candidates W-derived scaffolds.

The *A.* sp. *Kazakhstan* genome was assembled from 2 male short-read libraries with Megahit (v1.1.4, Li *et al.* 2015) and then scaffolded using SOAPdenovo-fusion (SOAPdenovo2 version 2.04, Luo *et al.* 2012).

BUSCO (version 5.2.2, Manni *et al.* 2021) was used to assess the completeness of the genomes generated in this study and the 2 previously published *A. franciscana* genomes in the genome mode with the arthropoda dataset (arthropoda_odb10).

### Estimation of genomic coverage

The short genomic reads were mapped to the genome using bowtie2 (version 2.4.4, Langmead and Salzberg 2012). The uniquely mapped reads were then extracted from the output sam files using grep (grep -vw “XS:i”). SOAP.coverage (version 2.7.7, Luo *et al.* 2012) was then used to calculate the coverage for each library either using 10,000-bp windows (*A. sinica*) or per scaffold (other species).

### Mapping of *A. franciscana* markers to the *A. sinica* genome

The sequences of the *A. franciscana* SLAF markers were obtained from Han *et al.* (2021), and the left and right pairs of each marker were mapped to the *A. sinica* male genome separately using pblat (Wang and Kong 2019). Only the mapping location with the largest match score was kept for each marker.

### Mapping of the *A. franciscana* and *A.* sp. *Kazakhstan* genomes to the new *A. sinica* assembly

We aligned the *A. sinica* published transcriptome (Huylmans *et al.* 2021) to both the *A. franciscana* and to the *A.* sp. *Kazakhstan* genomic scaffolds using blat (Standalone BLAT v. 36x2, Kent 2002). For each transcript, we kept only the mapping location with the highest score in each genome (using the customized script 1-besthitblat.pl). When multiple transcripts overlapped by more than 20 bp on the genome, only the transcript with the highest mapping score was kept (2-redremov_blat_v2.pl). We then used the location of the transcripts on the *A. sinica* genome to infer the location of the *A. franciscana* and *A.* sp. *Kazakhstan* scaffolds based on the transcripts they carried (AssignScaffoldLocation.pl). This script uses a majority rule to assign each scaffold to a chromosome, and then the mean location of genes on that scaffold to infer its final coordinate on the chromosome. All scripts are available on our git page.

### *F*_ST_ between male and female populations

RNA-seq reads from 10 pooled *A. sinica* males and 10 pooled *A. sinica* females (from Huylmans *et al.* 2021), sampled from head, thorax, and gonads, were trimmed with Trimmomatic (Bolger *et al.* 2014) and pooled by sex, and mapped separately to the male *A. sinica* reference genome using STAR (Dobin *et al.* 2013) with default parameters.

The resulting alignments with MAPQ score lower than 20 were filtered out and the remaining alignments were sorted using samtools view and sort functions (Li *et al.* 2009). Next, a pileup file of male and female alignments was produced using the samtools-mpileup function. Finally, we used scripts from the Popoolation2 package (Kofler *et al.* 2011) to calculate *F*_ST_. The mpileup file was reformatted with the Popoolation2 mpileup2sync.pl script, and the resulting synchronized file was used as an input for fst-sliding.pl script. *F*_ST_ between male and female populations was calculated for windows of 1,000 nucleotides, using the fst-sliding.pl script with following options –suppress-noninformative –min-count 3 –min-coverage 10 –max-coverage 200 –min-covered-fraction 0.5 –window-size 1,000 –step-size 1,000 –pool-size 10.

We applied the same pipeline to estimate male:female *F*_ST_ using head and gonad RNA-seq samples obtained from 10 males and 10 females of *A. franciscana* (from Huylmans *et al.* 2019). The resulting *F*_ST_ values were plotted based on the inferred location of the genomic scaffolds along the *A. franciscana* chromosomes (section *Mapping of the A. franciscana and A. sp. Kazakhstan genomes to the new A. sinica assembly*).

### Strata identification

**Fig. 1.**
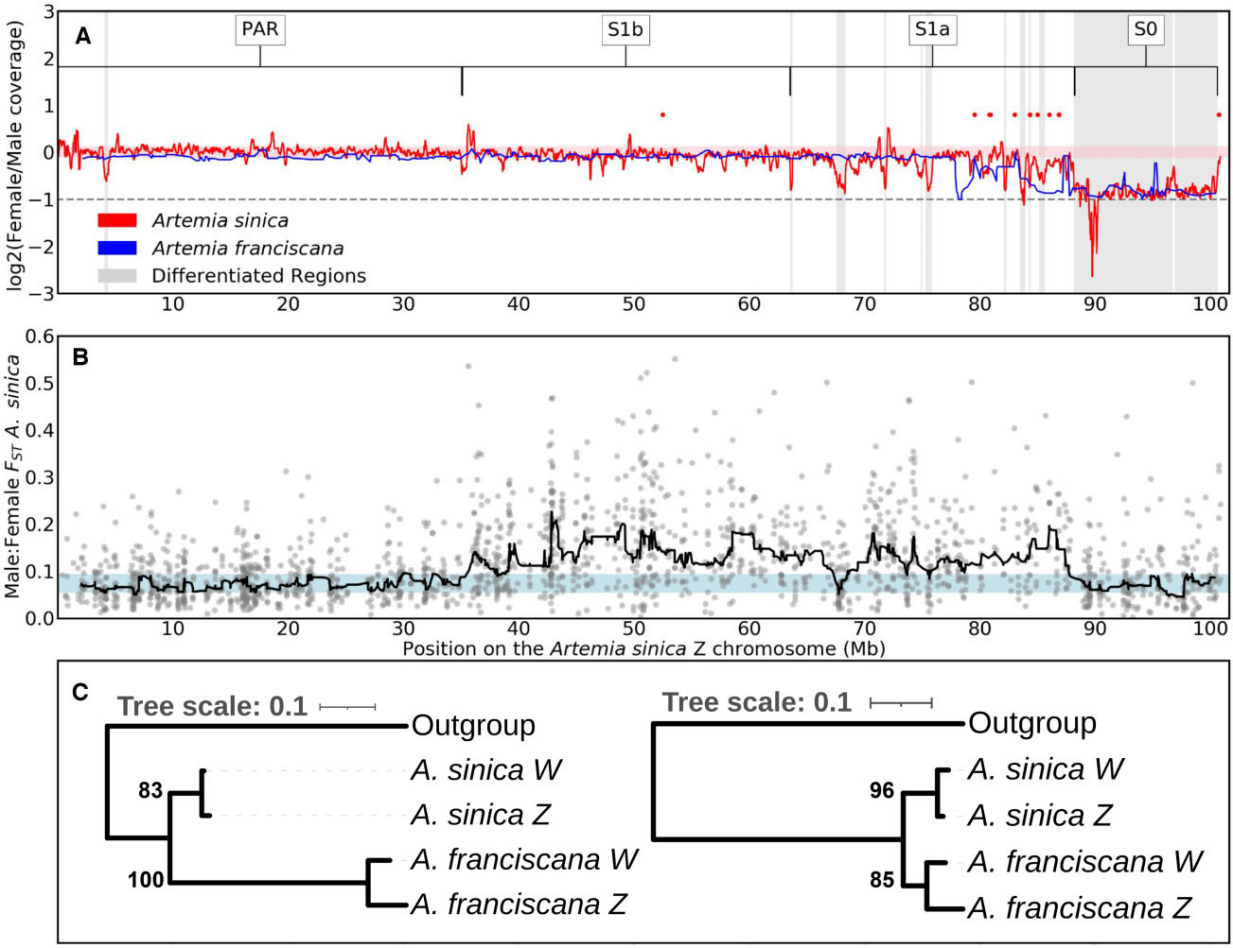
A shared sex-linked region on the ZW pair. a) Patterns of female/male coverage in *A. franciscana* and *A. sinica*. A rolling median of 20 windows (of 10 kb each) is shown in red for *A. sinica*, and a rolling median of 5 scaffolds is shown in blue for *A. franciscana*. The light pink shaded area highlights the region between the 5th- and 95th-percentiles of the rolling median of coverage for autosomes of *A. sinica*. The horizontal dashed line at −1 signifies the expected 2-fold reduction in coverage in females compared to males of fully differentiated regions. The inferred differentiated regions of *A. sinica* are highlighted in gray, and the putative strata are defined above, along with the putative pseudoautosomal region (PAR). The red dots are the locations of the W-candidates shared between *A. sinica* and *A. franciscana*. b) Male:female *F*_ST_ along the putative chromosome Z. The dots are *F*_ST_ calculated for 1-kb bins, and the line is the rolling median computed in sliding windows of 30 consecutive 1,000 nucleotide bins. The shaded area highlights the region between the 5th- and 95th-percentiles of the rolling median of *F*_ST_ for autosomes. c) Phylogenetic trees for 2 examples of the W-candidates shared between *A. sinica* and *A. franciscana* (red dots in a) and their putative Z homologs. *B. lindahli* is used as the outgroup.

ZW strata were identified based on the *A. sinica* coverage and *F*_ST_ analyses. First, we detected differentiated regions as any region where the Log2(female/male coverage) dropped below [median(autosomal windows) − 0.5)] for 10 consecutive 10-kB windows; each differentiated region was extended along the chromosome as long as Log2(female/male coverage) did not rise above that threshold for 10 consecutive windows (regions shaded in gray in Fig. 1). The 2 largest differentiated regions were nearly adjacent on the distal end of chromosome 1, and the whole region encompassing them was classified as S0 (no genes were found in the small undifferentiated region between them, such that including it in the S0 did not affect downstream analyses). We used a similar approach to detect regions of increased male:female *F*_ST_. In this case, only sparse information along the chromosome was obtained [as RNA-seq only provides single-nucleotide polymorphisms (SNPs) for genic regions], and many 1-kb bins were empty. We selected only informative bins and inferred an *F*_ST_ rolling median for 30 bins at a time (the median coordinate for the bins was similarly used as the coordinate for the resulting window). High *F*_ST_ regions were called when 10 consecutive rolling windows were above the 95%-percentile of autosomal windows, and these regions were extended along the chromosome until 10 consecutive windows were below this threshold. This yielded 3 nearly adjacent high *F*_ST_ sections (35.3–38.2, 38.4–67.3, and 68.5–87.7 MB), and the region encompassing them (35.3–87.7 MB) was classified as S1. S1 was further divided into S1a, which showed drops in female:male coverage, and S1b, which did not. The coordinate of the beginning of the first differentiated region within S1 was used as the boundary between them.

### Identification of candidate W-genes with k-mer analysis

We used a k-mer-based subtraction approach (Elkrewi *et al.* 2021) based on the tools included in the BBMap package (Bushnell 2014) on male and female genomic and RNA-seq data from *A. franciscana* and *A. sinica*. The pipeline was applied to each species separately. In *A. sinica*, 2 male and 2 female DNA libraries and 2 whole body RNA-seq replicates for each sex were used (Supplementary Tables 1 and 2). In *A. franciscana*, the analysis was performed using 1 male and 1 female DNA libraries and pools of 2 RNA-seq replicates of heads and gonads for each sex, along with 1 whole body male and female RNA-seq libraries (SRR14598203 and SRR14598204).

First, the shared 31-mers between the female DNA and RNA libraries were identified, and then any k-mers matching male libraries were removed. Female RNA-seq reads containing these female-specific k-mers [with minimum k-mer fraction of 0.6 (mkf = 0.6)] were retrieved and assembled using Trinity (Grabherr *et al.* 2011), and the perl script from the Trinity package (get_longest_isoform_seq_per_trinity_gene.pl) was used to keep only the longest isoform. The male and female genomic libraries were mapped to the assembled transcripts using Bowtie2 (Langmead and Salzberg 2012, p. 2), and candidates with a sum of female perfect matches ≤8 and a ratio of sum-of-females/sum-of-males ≤2 were removed. The final set consisted of 402 transcripts in *A. franciscana* and 319 in *A. sinica*.

### Mapping of W candidates to the *A. sinica* genome

The *A. sinica* and *A. franciscana* candidate W-derived transcripts were mapped to the *A. sinica* genome assembly with Parallel Blat (Wang and Kong 2019) with a translated query and database, and a minimum match score of 50. Only alignments with match scores above 100 were considered, and the mapping location with the strongest match score was considered for each transcript.

### Transcriptome assemblies and expression analysis

The *A. sinica* male transcriptome was assembled from 2 replicates of male whole body RNA-seq data (Huylmans *et al.* 2021) using Trinity (Grabherr *et al.* 2011) in 2 different modes: denovo and genome-guided. The 2 assemblies were concatenated and then filtered using the tr2aacds.pl script from EvidentialGene (Gilbert 2019). The transcriptome was annotated with the Pannzer annotation server (Törönen *et al.* 2018), and mapped to the *A. sinica* genome using the same procedure as described in the section *Mapping of W candidates to the A. sinica genome*.

For the expression analysis, only the first isoform was kept for each gene, and only transcripts longer than 500 bp were used in the analysis. The RNA-seq reads from the *A. sinica* heads, gonads, and thoraces of males and females (Huylmans *et al.* 2021) were mapped to the curated transcriptome and gene expression levels (in Transcripts per million, TPM) were obtained using Kallisto (version 0.46.2, Bray *et al.* 2016). Quantile normalization was done using NormalyzerDE (Willforss *et al.* 2019).

Two different *A. franciscana* de novo transcriptome assemblies were made using Trinity. The first using pooled RNA-seq reads from male heads and testes [2 replicates each (Huylmans *et al.* 2019)], and the second using the published whole-body male RNA-seq library (SRR14598203, Jo, Lee, Choi, Kim, Lee, and Park 2021). The 2 assemblies were concatenated and then filtered using the tr2aacds.pl script from EvidentialGene, and mapped to the *A. sinica* genome using the same procedure as described in section *Mapping of W candidates to the A. sinica genome*.

### Phylogenetic trees

The W candidates of *A. sinica* and *A. franciscana* were mapped reciprocally to each other using pblat (v. 36x2 with default parameters, Wang and Kong 2019), and reciprocal best hits were considered shared candidates. The W candidates of the 2 species were further mapped to their respective uncollapsed male transcriptome assemblies (see previous section) with pblat (Wang and Kong 2019) with a translated query and database, and a minimum match score of 50. The transcripts with the highest mapping score to the W candidates were used as the putative Z homologs in their respective species.

The *Branchinecta lindahli* transcriptome (Schwentner *et al.* 2018) was downloaded from the Crustacean Phylogeny dataset on Harvard Dataverse (https://doi.org/10.7910/DVN/SM7DIU). *B. lindahli* homologs of shared W-candidates were obtained by mapping the putative Z homologs of both species to the *B. lindahli* transcriptome using pblat (-minScore = 50 -t = dnax -q = dnax) and retrieving the transcript with highest alignments score (using the customized script 2-besthitblat.pl). A transcript was considered a homolog if it mapped to at least one of the putative Z homologs of the 2 species, and when the 2 Z homologs mapped to different outgroup sequences, both outgroup sequences were retrieved and used to make 2 different alignments.

The shared W candidates of *A. sinica* and *A. franciscana*, their Z homologs, and the outgroup sequences were aligned using MAFFT (version v7.487, with the options “mafft –adjustdirection INPUT > OUTPUT,” Katoh *et al.* 2002). The resulting alignments were fed to phylogeny.fr (Dereeper *et al.* 2008), where the alignment was curated using Gblocks (Talavera and Castresana 2007), and the phylogenetic tree was constructed using PhyML (Guindon *et al.* 2010). Trees were made only for sequences where the number of overlapping positions after Gblocks was longer than 200 bp. In the 4 instances where the curated alignment length with the outgroup was shorter than 200 bp, we tried aligning the sequences without the outgroup. For the 2 cases where the resulting alignment length was longer than or equal 200 bp, unrooted trees were made. The trees were then downloaded in the Newick format and visualized using itol.embl.de (Letunic and Bork 2019).

### Heterozygosity analysis in asexual female and rare male

Illumina genomic sequencing was performed on a rare male and its asexual sister (both derived from an Aibi Lake *A. parthenogenetica* lineage), yielding around 115 million paired-end reads with a length of 125 nucleotides for each sample. The reads were quality- and adapter- trimmed with Trimmomatic-0.36 (Bolger *et al.* 2014) and mapped to the draft *A.* sp. *Kazakhstan* genome assembly using STAR v.2.6.0c (Dobin *et al.* 2013) with default settings.

We indexed the reference *A.* sp. *Kazakhstan* genome using SAMtools v.1.10 (Li *et al.* 2009), called the SNPs from BAM alignments with BCFtools v.1.10.2 (Li *et al.* 2009), then removed indels, filtered for quality of reads over 30 and coverage over 5 and below 100 with VCFtools v.0.1.15 (Danecek *et al.* 2011), and removed multiallelic sites with BCFtools.

We calculated the fraction of SNPs that lost heterozygosity in the rare male DNA in comparison with the asexual sister DNA. It was calculated and visualized in 500-kb bins for each chromosome.

### Crossing design to identify the asexuality locus

We designed a backcross to investigate the loci controlling asexuality. An asexual female from Aibi Lake produced a rare male. We crossed this male with an inbred female from the closest related sexual species, *A.* sp. *Kazakhstan*. This produced asexual females and males in the F1 generation. We then backcrossed 12 males from the F1 to sexual females from the same inbred line of *A.* sp. *Kazakhstan*. Of these, 6 crosses produced offspring, yielding a total of 84 males, 5 asexual females, and 96 putatively sexual females (those that did not reproduce asexually for 133 days after the crosses were set up). The 5 asexual females and 10 control females were used individually for DNA extractions with the Qiagen DNeasy Blood & Tissue kit. The control females came from the same crosses (i.e. had the same F2 father and *A.* sp. *Kazakhstan* mothers) as the asexual females, but were otherwise selected randomly. Illumina short-read sequencing was then performed as described in the section *DNA short- and long-read sequencing*.

### Analysis of backcross between the Aibi Lake rare male and *A.* sp. *Kazakhstan* females

We sequenced 5 asexual females and 10 putatively sexual females from the F2 generation. This resulted in an average of 101 million reads per asexual female and 50 million reads per putatively sexual female. We first used SEQTK v1.2 (https://github.com/lh3/seqtk) to randomly select a subset of reads from each asexual sample to match the number of reads of the smallest sample (to avoid biasing allele estimates toward high-coverage individuals). We removed adaptors and trimmed reads using Trimmomatic v0.39 (Bolger *et al.* 2014). We then aligned the resulting paired-end reads to the genome using Bowtie2 v2.4.4 (Langmead and Salzberg 2012). SAM files were converted to BAM files and sorted in Samtools v.1.13 (Li *et al.* 2009).

For our pooled analyses, we merged BAM files into a pooled asexual BAM file and a pooled putatively sexual BAM, and created a mpileup file in Samtools v.1.13. We then used Popoolation2 (Kofler *et al.* 2011) to call *F*_ST_ for both individual SNPs and in 1-kb windows. We used *F*_ST_ computed for 1-kb windows to visualize *F*_ST_ across the genome in a Manhattan plot in the R package qqman (Turner 2018). We computed rolling medians in sliding windows of 101 consecutive SNPs on each linkage group using the rollmedian function from the package zoo (Zeileis and Grothendieck 2005) in R v.4.0.3. To identify regions of elevated *F*_ST_ on individual chromosomes, we computed 95% confidence intervals by sampling rolling medians of 101 consecutive SNPs across the genome 1,000 times.

For our individual-based analyses, we similarly used SEQTK v1.2 to randomly select a subset of reads from each asexual sample to match the highest coverage found in an F2 control female (to avoid biases caused by the much larger number of reads obtained for the F2 asexuals than for the controls). We then mapped reads from all F2 individuals to the *A.* sp. *Kazakhstan* genome using BWA mem v0.7.17 (Li and Durbin 2009) with default parameters. DNA reads from the rare male and its *A. parthenogenetica* sister, and from 2 *A.* sp. *Kazakhstan* individuals, were also subsetted and mapped. The resulting BAM alignments were sorted with samtools v1.14 (Li *et al.* 2009), and used to call SNPs with the mpileup function of BCFtools v1.14 (Li 2011). The VCF file was filtered with VCFtools v0.1.17 (Danecek *et al.* 2011) for minimum and maximum depth (4 and 50), minimum quality score (30), and minimum minor allele frequency (0.1). Only SNPs for which the 2 *A.* sp. *Kazakhstan* had a genotype of 0/0, and the 2 *A. parthenogenetica* individuals 1/1, were kept for further analyses. We computed *F*_ST_ between the F2 asexual and control females using the function –weir-fst-pop in VCFtools for 10-kb windows. We then inferred ancestry of each genomic scaffold in every sample (i.e. whether they were homozygous for the *A.* sp. *Kazakhstan* haplotype, or carried a copy of the *A. parthenogenetica* haplotype as well) using the customized script Chromopaint.pl (available on our git page). The *A.* sp. *Kazakhstan* genomic scaffolds were assigned to a location on the *A. sinica* genome as before. Scaffolds with more than 10 informative SNPs, and >80% 0/1 or 1/1 SNPs were considered to be heterozygous for the *A.* sp. *Kazakhstan* and *A. parthenogenetica* haplotypes, whereas scaffolds with >80% 0/0 were considered to have only *A.* sp. *Kazakhstan* ancestry (only 5–9% of scaffolds fell in between and could not be classified in each individual).

## Results

### The ZW pair is shared by American and Eurasian *Artemia*

Two genome assemblies and a high-density linkage map are currently available for the American *A. franciscana* (Jo, Lee, Choi, Kim, Lee, Lee, *et al.* 2021; Han *et al.* 2021; De Vos *et al.* 2021), but resources for the Eurasian clade are more limited, with only an *A.* sp. *Kazakhstan* draft genome assembly recently described in Boyer *et al.* (2022). The median dS (the number of synonymous substitutions per synonymous site) between the 2 clades is ∼0.2. We assembled a male genome of *A. sinica* using PacBio long reads (∼30×) and Hi-C Illumina reads (1.5*e12 reads), yielding 1,213 scaffolds with an N50 of 67.19 Mb (Supplementary Fig. 2 and Table 3) and a total length of 1.7 Gb; 85% of the sequences get assigned to one of the 21 largest scaffolds (which corresponds to the expected number of chromosomes, Sainz-Escudero *et al.* 2021). The strong diagonal in the heatmap of the Hi-C contact matrix (Supplementary Fig. 3) supports the high quality of our assembly, as does our BUSCO score of 91.8%. This chromosome-level assembly represents an improvement over existing *Artemia* genomes, which have N50 values of 27–112 kb, and BUSCO scores of 68.3–86.9% (Jo, Lee, Choi, Kim, Lee, Lee, *et al.* 2021; De Vos *et al.* 2021; Boyer *et al.* 2022; Supplementary Fig. 4).

Our earlier analysis of female and male genomic coverage in *A. franciscana* had uncovered a small region of reduced female coverage, consistent with full differentiation of the Z and W chromosomes (Huylmans *et al.* 2019). To investigate whether ZW differentiation was also present in *A. sinica*, we first estimated male and female coverage along each chromosome. Consistent with *A. franciscana*, only a small genomic region on chromosome 1 had decreased female/male coverage (Fig. 1a; Supplementary Fig. 5 for all chromosomes), showing that chromosome 1 is the Z chromosome. To check for homology with the *A. franciscana* differentiated region, we mapped the scaffolds from the published *A. franciscana* genome (Jo, Lee, Choi, Kim, Lee, Lee, *et al.* 2021) to the new *A. sinica* assembly based on their shared gene content, and plotted the coverage values that we had previously estimated (Huylmans *et al.* 2019) based on the *A. sinica* coordinates. Figure 1a shows that the 2 differentiated regions largely overlap, supporting the ancestry of the pair of sex chromosomes; we name this shared region stratum 0 (S0). In the *A. franciscana* linkage map (Han *et al.* 2021), LG6 was identified as the sex chromosome. To further verify the homology between the ZW pairs of the 2 species, we mapped the genetic markers used by Han *et al.* (2021) to our *A. sinica* assembly. As expected, the vast majority of LG6 markers for which we could infer a location mapped to our chromosome 1 (Supplementary Fig. 6). We also produced an assembly based on *A. sinica* female long PacBio reads, which contains a substantial amount of scaffolds with excessive female coverage, consistent with W-linkage (Supplementary Fig. 7).

### Convergent loss of ZW recombination

To identify parts of the sex chromosomes that no longer recombine, but are still similar enough that W-derived reads still map to the Z, we used previously published RNA-seq data for *A. sinica* (Huylmans *et al.* 2021), obtained from 10 males and 10 females, to estimate *F*_ST_, a measure of genetic differentiation, between the 2 sexes. Genetic variants found exclusively on the W increase the level of female–male differentiation, and young nonrecombining regions can be detected through their high male:female *F_S_*_T_ (Palmer *et al.* 2019; Vicoso 2019; Gammerdinger *et al.* 2020). Such an increase in male:female *F*_ST_ is not expected for the highly differentiated S0, since W-derived reads do not map to this part of the Z-chromosome. Figure 1b shows that a large region (∼52 Mb) has *F*_ST_ values systematically above the 95th-percentile of autosomes, consistent with recent loss of recombination in *A. sinica*. We call this region stratum 1 (S1), but further divide it into S1a, which shows localized drops in female:male coverage (gray-shaded regions in Fig. 1a), and S1b, for which no coverage differences are observed (Fig. 1a), and which may still undergo some recombination. The distal end of S1a has reduced female:male coverage in *A. franciscana*, and an *F*_ST_ analysis in this species yielded increased male:female *F*_ST_ from ∼60 to 85 MB (see Supplementary Fig. 8), showing that at least part of this region has also stopped recombining in the American lineage.

Given the substantial distance between the Eurasian and American lineages, we hypothesized that the loss of recombination in S1 had occurred independently in the 2 clades. To test this, we used a k-mer-based pipeline combining male and female DNA and RNA short reads (Elkrewi *et al.* 2021) to identify putative W-derived transcripts. This yielded 402 transcripts in *A. franciscana* and 319 in *A. sinica*. Of those that mapped to the genome, 180 out of 306 (59%) *A. sinica* transcripts and 168 out of 355 (47%) *A. franciscana* transcripts mapped to chromosome 1 (Z) of *A. sinica*, a higher proportion than the overall 7% of genes that map to this chromosome, confirming the validity of the approach (since we expect many W-linked genes to have a close homolog on the Z). Few of these candidate W genes mapped to the putative ancestral sex-linked region (16W-linked genes in *A. sinica*, compared to 84 Z-linked genes, and 7 vs 91 in *A. franciscana*, Supplementary Table 4), consistent with substantial degeneration of this part of the W-chromosome. To find genes present on the W-chromosomes of both species, we selected reciprocal best hits between the 2 sets of W candidates. Most of the candidates that were found in both species (10 out of 15) mapped to the putative S1a region. We made phylogenetic trees using each pair of homologous W-genes and their Z-linked homologs (obtained from a male-only transcriptome assembly), to infer whether these genes were W-linked before the split of the 2 clades. The closest homolog in the transcriptome of the distantly related fairy shrimp *B. lindahli* (Schwentner *et al.* 2018) was used as an outgroup sequence, when one could be detected. Figure 1c shows the resulting phylogenetic trees for 2 of the shared W-linked genes and their Z-homologs, while phylogenies for all candidates are provided in Supplementary Fig. 9. In every case, ZW homologs clustered by species rather than by chromosome, confirming that loss of recombination occurred independently and convergently for this region in the American and Eurasian lineages.

### Dosage compensation of the Z-specific region

**Fig. 2.**
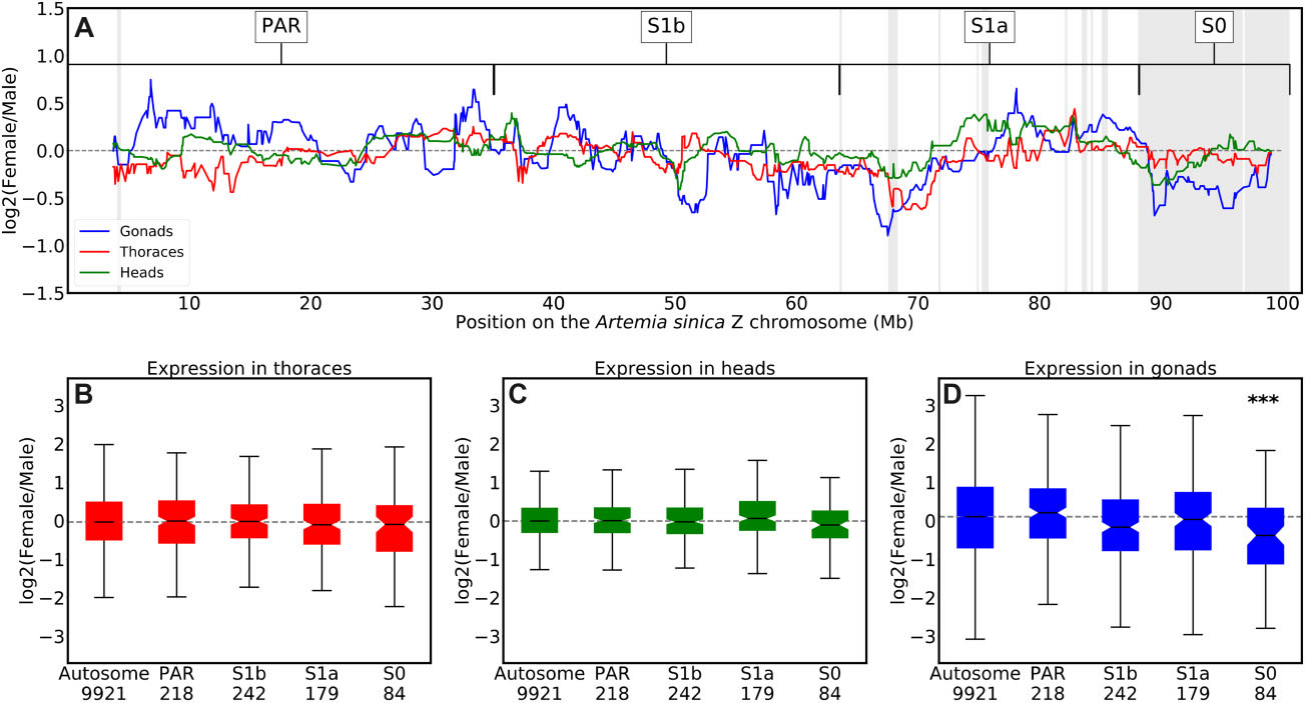
Dosage compensation of the Z-chromosome. a) The log-transformed ratio of female to male expression along the Z chromosome in heads, gonads, and thoraces (computed as the rolling median in sliding windows of 30 consecutive genes). Shaded areas represent the differentiated regions identified in the coverage analysis, and the putative strata are denoted above, along with the putative pseudoautosomal region (PAR). The dashed horizontal line is at zero. The distribution of log-transformed ratio of female to male expression for the autosomes and the different regions of the Z chromosome in thoraces (b), heads (c) and gonads (d). The number of genes in each of the different regions is indicated underneath the *x*-axis labels. A Wilcoxon rank sum test was used to assess the significance of the difference between the expression of the autosomes and the different regions of the Z chromosome, with a Bonferroni correction for the 4 comparisons performed in each tissue. ***P-value ≤ 0.001.

Many female-heterogametic species lack a chromosome-wide mechanism of dosage compensation, and investigating the few cases that have it may shed light on the difference between ZW and XY systems. Earlier work suggested that the Z-specific region of *A. franciscana* was compensated (Huylmans *et al.* 2019), but misidentification of genes in the sex-linked region (as the genome was fragmented) could have hidden differences between chromosomes. We repeated this analysis using RNA-seq data from thorax, head, and gonad of *A. sinica* (Huylmans *et al.* 2021). We first assembled a male transcriptome from all pooled male reads available for this species (to avoid hybrid assemblies of Z and W homologs, see Supplementary Fig. 10 for a BUSCO assessment), mapped it to the male genome assembly, and estimated expression for each sample (in TPM). In somatic tissues, the female:male ratio is similar for the autosomal genes and S0 genes (*P* = 0.2 and *P* = 0.6 in heads and thoraces, Bonferroni-corrected Wilcoxon tests, Fig. 2b and c), confirming that dosage compensation is active in this clade. A significant shift toward male-biased expression can be observed for the S0 in gonads (*P* = 0.0007, Bonferroni-corrected Wilcoxon test, Fig. 2d). A table with nominal *P*-values for each comparison is provided in Supplementary Table 5.

### The sex chromosomes of asexual females and the genetic origin of rare males

**Fig. 3.**
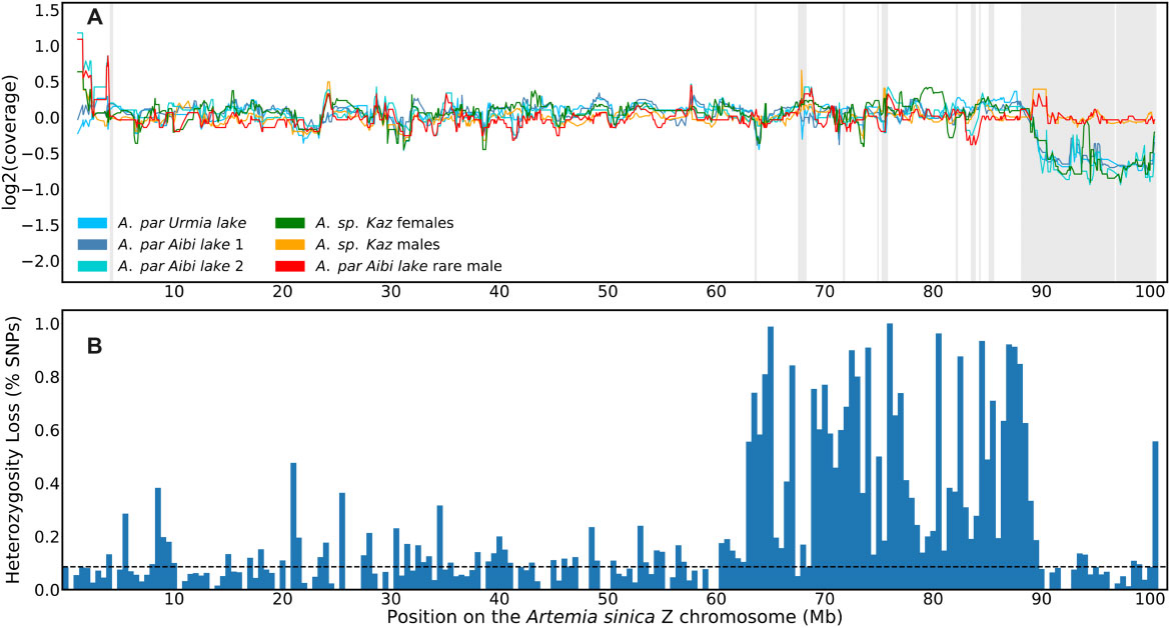
The sex chromosomes of sexual and asexual individuals. a) Coverage patterns in *A.* sp. *Kazakhstan* male and female samples, in 3 asexual females, and in a rare male derived from an asexual lineage from Aibi Lake. Shaded areas represent the differentiated regions of the *A. sinica* ZW pair. b) The fraction of SNPs that lost heterozygosity on the rare male Z chromosome relative to its asexual sister in bins of 500 kb. The dashed line represents the average loss of heterozygosity for autosomes.

In order to characterize the ZW pair of asexual females, we first obtained a draft genome assembly of the closely related sexual species *A.* sp. *Kazakhstan* from illumina short reads (Supplementary Table 6), and estimated genomic coverage using 2 female and 2 male samples of this species. The genomic scaffolds were mapped to the *A. sinica* genome based on their gene content, and median coverages of male and female *A.* sp. *Kazakhstan* individuals were plotted along the *A. sinica* Z chromosome using a sliding window of 10 scaffolds (green and yellow lines in Fig. 3a). As expected, an approximately 2-fold drop in female coverage was observed in a similar region to that found in *A. sinica* (marked by gray shading), whereas the male harbored high genomic coverage throughout the chromosome, consistent with the presence of the same pair of sex chromosomes in this lineage (a similar pattern was observed in *A. urmiana*, Supplementary Fig. 11). We used the *A.* sp. *Kazakhstan* draft genome to map genomic reads derived from 3 closely related asexual females (1 from the Lake Urmiana-derived population and 2 from a population derived from Aibi Lake cysts). In every case, the patterns of coverage were very similar to those of the *A.* sp. *Kazakhstan* sexual female, confirming that asexual females carry the same pair of ZW chromosomes.

Diploid *A. parthenogenetica* likely reproduce through central fusion automixis, a modified form of meiosis that preserves heterozygosity in the genome except at distal ends of chromosomes when recombination has occurred (Nougué *et al.* 2015). Boyer *et al.* (2022) recently showed that *Artemia* rare males can be produced by ZW recombination events at variable locations near the sex-determining locus. We obtained a rare male from an *A. parthenogenetica* line from Aibi Lake (which we use in the next section to explore the transmission of asexuality). To test whether it arose through ZW recombination or other chromosomal changes, we first compared patterns of genomic coverage to those of females. No reduced coverage was observed along the Z-chromosome, arguing against the loss of a sex chromosome. We further called SNPs in the rare male and in its sister (marked as A. par. Aibi lake 2 in Fig. 3a) and estimated the proportion of heterozygous SNPs present in the asexual female that were lost in the rare male. Loss of heterozygosity was detected throughout the distal half of the Z-chromosome (Fig. 3b; Supplementary Fig. 12), confirming that a large part of the W was replaced by its Z homologous region. A substantial loss of heterozygosity was also found at the beginning of chr 13, and smaller regions of decreased heterozygosity may be present at the ends of several chromosomes (Supplementary Fig. 12). Taken together, these results support central fusion automixis as the mode of reproduction of *A. parthenogenetica*, and rare ZW recombination as the source of the Aibi Lake rare male (Nougué *et al.* 2015; Boyer *et al.* 2022).

### The Z chromosome likely contributes to the transmission of asexuality

In order to find possible loci responsible for the spread of asexuality in brine shrimp, we crossed the rare male described in the previous section and a sexual female from *A.* sp. *Kazakhstan* (Supplementary Fig. 13). This produced 22 asexual females and 24 males in the F1; 1 additional female died without producing offspring asexually. The presence of asexual females in the F1 shows that the locus controlling asexuality in this lineage works in a dominant manner, unlike what was first observed in Maccari *et al.* (2014), but consistent with the recent experiments of Boyer *et al.* (2021). The fact that almost all females produced offspring without mating further suggests that the locus was likely present on both copies of the genome of the original rare male. We then backcrossed 12 males from the F1, which should only carry 1 copy of the locus/loci controlling asexuality, with females from an *A.* sp. *Kazakhstan* inbred line (of these only 6 yielded progeny). The resulting F2 generation consisted of 84 (∼45%) males, 5 (∼3%) asexual females, and 96 (∼52%) females that did not produce asexually 133 days after the crosses were set up (44 individuals died before sexing was possible and are not included in the counts; see counts for individual crosses in Supplementary Table 7). We presume that most of these are sexual females for our analyses, but some could have reproduced asexually had the experiment been continued longer.

**Fig. 4.**
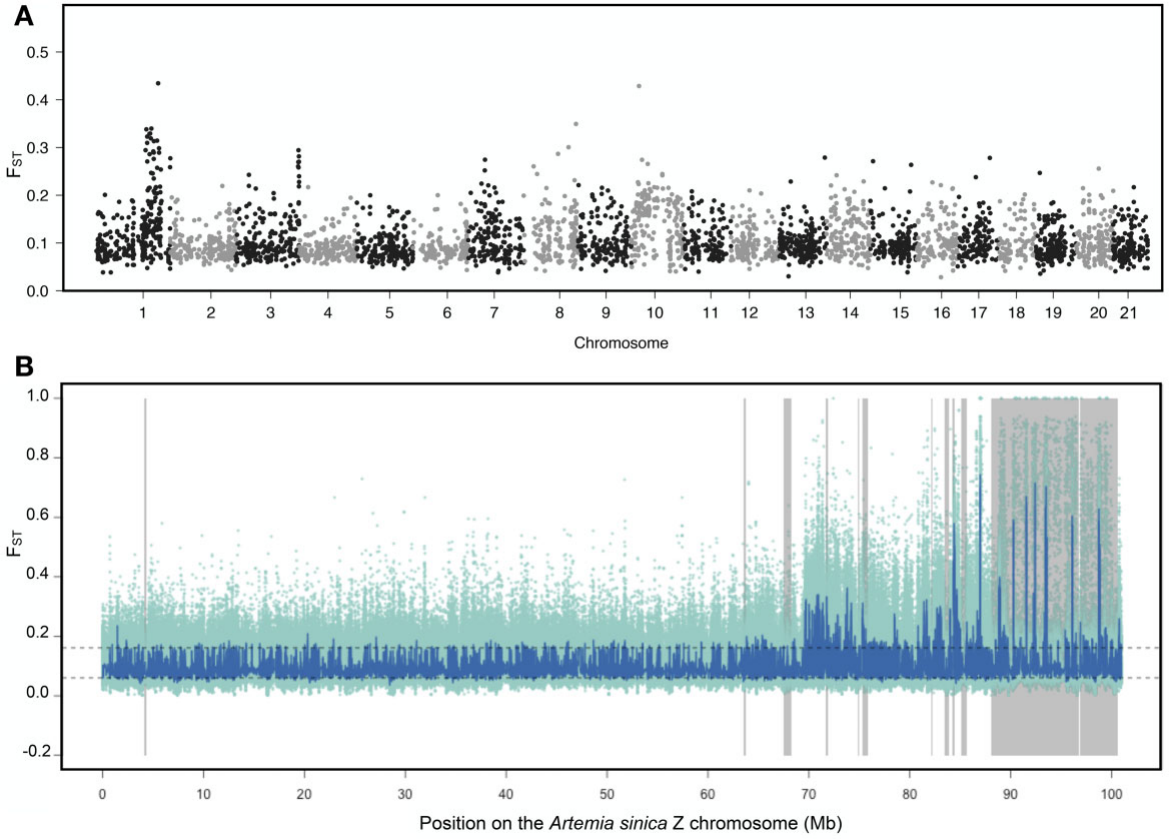
Elevated F_ST_ between sexual and asexual females localizes to the Z chromosome. a) Manhattan plot of *F*_ST_ estimated for 1 kb sliding windows between asexual and sexual females across the genome. b) *F*_ST_ across chromosome 1. *F*_ST_ is shown for individual SNPs in light green dots, and the dark line shows the rolling median for 101 SNPs. The dashed lines represent the 2.5% and 97.5% percentiles of autosomal rolling medians. Areas shaded in gray represent the differentiated regions of the *A. sinica* ZW pair.

We produced whole-genome resequencing data for the 5 F2 asexual females and, as a control, 10 F2 putatively sexual females. These were first pooled into an asexual pool and a putatively sexual pool, and we used Popoolation2 to compute *F*_ST_ between these 2 pools of females. While a few small peaks of *F*_ST_ are found on the autosomes (Fig. 4a), the strongest signal comes from the distal end of the Z chromosome (Fig. 4b). We further predicted that loci underlying asexuality should have been inherited from the original rare male by all the F2 asexual females, but not by (all) control females. To test this, we mapped all DNA samples individually to the *A.* sp. *Kazakhstan* genome. We also mapped the original rare male and its *A. parthenogenetica* sister, and 2 *A.* sp. *Kazakhstan* individuals, in order to select SNPs that were alternatively fixed between the 2 lineages. We used these informative SNPs to re-estimate *F*_ST_ between F2 asexual and control females, and to infer which genomic regions were inherited from the rare male by each of the F2 individuals. Supplementary Figure 14 shows that we recover a region of high *F*_ST_ on the Z chromosome, and that all asexuals carry genetic material from the rare male in this region, as expected if it controls asexuality. In total, only 17 scaffolds with an assigned location on the *A. sinica* genome show ancestry patterns consistent with an asexuality locus (i.e. they show evidence of *A. parthenogenetica* ancestry in all asexual females, but not in all control females). Eleven are on the Z chromosome (vs 1 expected, *P* = 1.3e−20 with a chi-square test) and correspond to the region of high *F*_ST_, providing further support for a role of the Z chromosome in the transmission of asexuality. None of the other minor peaks of *F*_ST_ are in regions with ancestry patterns consistent with asexuality loci (Supplementary Fig. 14), although chromosome 16 contains 3 such loci (vs 0.9 expected, *P* < 0.01 with a chi-square test). The annotation of genes located in the Z-linked candidate locus did not yield any obvious candidates (the annotation for all transcripts is provided as a Supplementary Dataset).

## Discussion

The potential of Artemia brine shrimp as models for ZW chromosome evolution and comparative genomics in general was until recently hampered by a lack of genomic resources. The publication of 2 genomes for the American *A. franciscana* has already shed new light on how these charismatic organisms survive in their extreme environments (Jo, Lee, Choi, Kim, Lee, Lee, *et al.* 2021; De Vos *et al.* 2021), but no information on sex linkage was provided, and the lack of a close outgroup sequence (other than the distant Daphnia) made comparative analyses difficult. A draft genome was recently described for *A.* sp. *Kazakhstan* (Boyer *et al.* 2022), but there was limited power to assign scaffolds to the sex chromosomes or autosomes.

Here, we obtain the first chromosome-level assembly in the clade for the Eurasian brine shrimp *A. sinica* and characterize in detail the differentiated and undifferentiated regions of the ZW pair. By combining these results with those of a preliminary analysis in *A. franciscana* (Huylmans *et al.* 2019), we confirmed the putative evolutionary model for the ZW pair, with an ancient well-differentiated region that stopped recombining in the ancestor of the 2 lineages, and more recent “strata” arising in each lineage independently. The independent loss of recombination in American and Eurasian species provides a unique opportunity to investigate convergent changes that occur in early sex-chromosome evolution. In agreement with previous findings in *A. franciscana*, *A. sinica* males and females have similar somatic expression patterns of Z-linked genes in the differentiated region which strongly supports the presence of a mechanism of dosage compensation in this group. A significant male-bias in expression was found for S0 genes in the gonads. Such differences in the gonad have been found even in animals with well-characterized chromosome-wide mechanisms of dosage compensation, such as *Drosophila* (Meiklejohn *et al.* 2011) and silkworm (Huylmans *et al.* 2017). While compensation mechanisms may be absent or less active in the gonad (Meiklejohn *et al.* 2011), differences could also result from the unusual regulation of the sex chromosomes in the germline (Argyridou and Parsch 2018), where they are often inactivated or downregulated (Vibranovski *et al.* 2009). Currently, no tractable lab model exists for the early evolution of ZW chromosomes, and Z-chromosome dosage compensation is only understood in detail for the silkworm (Walters and Hardcastle 2011; Kiuchi *et al.* 2014; Katsuma *et al.* 2019; Rosin *et al.* 2022), making this an outstanding new model clade for investigating these topics.

Finally, we obtained several putative W-genes in both species using a k-mer-based analysis. Few of them mapped to the ancestral part of the W chromosome: only ∼20% of the Z-linked genes in this region have a W-homolog, suggesting that much of the ancestral gene content has been lost. All of the genes for which a W-homolog could be found in both *A. sinica* and *A. franciscana* mapped to younger strata of the ZW pair and appear to have become W-linked independently in the 2 lineages. If the ancestral sex-determination mechanism is still shared by the 2 species, this may suggest that the primary signal for sex determination is a dosage-dependent gene on the Z rather than a dominant female-determining gene on the W. However, it is also possible that the sex-determining gene is only expressed early in development and was missed by our analysis of adult tissues. Future studies of sex-specific expression throughout the life cycle and complete assemblies of the W chromosome of the 2 species will be necessary to shed light on sex determination in this group.

The proximity of *A. sinica* to the *A.* sp. *Kazakhstan* group, which contains both sexual and asexual populations, also allowed us to characterize sex-linked sequences in this group. First, we found that the sex chromosome pair is shared by all populations. We further confirmed that rare males in this group can be produced through the replacement of the W-specific region with its Z-counterpart, showing that the same mechanism is used for rare male production in *A. parthenogenetica* isolates from widely differing geographic origins (China in this study, Iran and France in Boyer *et al.* 2022). Finally, our backcrossing experiment points to a role of the sex chromosome pair in the spread of asexuality through rare males.

It should be noted that this experiment has several drawbacks. First, it is difficult to phenotype females as sexual or asexual, as the timing at which asexuals produce their first brood can vary (although they typically do so within 30 days of hatching, Amat *et al.* 2007). Furthermore, hybrid incompatibilities may stop females from producing viable offspring even if they carry the alleles encoding asexuality. The fact that less than 5% of females were asexual in the F2 suggests that the trait is polygenic, and/or that we are mistakenly classifying asexuals as putative sexuals. Finally, we only obtained a small number of backcrossed asexual females, which limits the power to infer causal loci.

Despite these drawbacks, the Z chromosome showing the strongest signal of differentiation between asexual and control females is intriguing, and in line with results in the pea aphid, which carries the asexuality locus on the X chromosome. In species where asexuality is triggered by an endoparasite such as Wolbachia, the acquisition of asexuality is thought to be driven by the transmission advantage gained by the female-transmitted parasite (since asexual reproduction leads to an all-female progeny). It is possible that an asexuality gene found on a Z chromosome similarly benefits from a transmission advantage. If rare males always arise through the replacement of the W-specific region with its Z homolog region, a Z-linked asexuality locus will be homozygous and therefore transmitted to all daughters in the F1 (and to all sons). More detailed studies of transmission of asexuality in this group and others with ZW and XY sex chromosomes will in the future shed light on the relationship between sex determination and the rise and spread of asexual reproduction under various sex-determining mechanisms.

## Supplementary Material

iyac123_Supplemental_Material

iyac123_Tables_S1_and_S2

## Data Availability

All genomic reads generated for this study are available at the NCBI short reads archive under Bioproject number PRJNA848277. The pipelines used to analyze the data are at https://git.ist.ac.at/bvicoso/zsexasex2021, and important processed data files such as the new *A. sinica* genome assembly are provided at https://doi.org/10.15479/AT:ISTA:11653. Supplemental material is available at *GENETICS* online.

## References

[iyac123-B1] Abatzopoulos TJ. The repeated emergence of asexuality, the hidden genomes and the role of parthenogenetic rare males in the brine shrimp Artemia. J Biol Res (Thessalon). 2018;25(7):7. doi:10.1186/s40709-018-0078-2. 29796384 PMC5960178

[iyac123-B2] Accioly IV , Cunha IMC, Tavares JCM, Molina WF. Chromosome banding in Crustacea. I. Karyotype, Ag-NORs, C banding and treatment with EcoRI, PstI and KpnI restriction endonucleases in *Artemia franciscana*. Biota Amaz. 2014;4(2):15–19.

[iyac123-B3] Amat F , Hontoria F, Navarro JC, Vieira N, Mura G. Biodiversity loss in the genus Artemia in the Western Mediterranean Region. Limnética. 2007;26(2):387–404.

[iyac123-B4] Argyridou E , Parsch J. Regulation of the X chromosome in the germline and soma of *Drosophila melanogaster* males. Genes. 2018;9(5):242. doi:10.3390/genes9050242. PMC5977182 29734690

[iyac123-B5] Arima Genomics. mapping_pipeline. Arima Genomics Inc., 2021. https://github.com/ArimaGenomics/mapping_pipeline.

[iyac123-B6] Aumer D , Allsopp MH, Lattorff HMG, Moritz RFA, Jarosch-Perlow A. Thelytoky in Cape honeybees (*Apis mellifera capensis*) is controlled by a single recessive locus. Apidologie. 2017;48(3):401.

[iyac123-B7] Bachtrog D. Y-chromosome evolution: emerging insights into processes of Y-chromosome degeneration. Nat Rev Genet. 2013;14(2):113–124. doi:10.1038/nrg3366. 23329112 PMC4120474

[iyac123-B8] Bachtrog D , Mank JE, Peichel CL, Kirkpatrick M, Otto SP, Ashman T-L, Hahn MW, Kitano J, Mayrose I, Ming R, et al; Tree of Sex Consortium. Sex determination: why so many ways of doing it? PLoS Biol. 2014;12(7):e1001899. doi:10.1371/journal.pbio.1001899. 24983465 PMC4077654

[iyac123-B9] Baxevanis AD , Kappas I, Abatzopoulos TJ. Molecular phylogenetics and asexuality in the brine shrimp Artemia. Mol Phylogenet Evol. 2006;40(3):724–738. doi:10.1016/j.ympev.2006.04.010. 16753307

[iyac123-B10] Beukeboom LW , Perrin N. The Evolution of Sex Determination. Oxford: Oxford University Press, 2014.

[iyac123-B11] Bolger AM , Lohse M, Usadel B. Trimmomatic: a flexible trimmer for Illumina sequence data. Bioinformatics. 2014;30(15):2114–2120. doi:10.1093/bioinformatics/btu170. 24695404 PMC4103590

[iyac123-B12] Bowen ST. The genetics of *Artemia salina*. III. Effects of X-irradiation and of freezing upon cysts. Biol Bull. 1963;125(3):431–440. doi:10.2307/1539357.

[iyac123-B13] Boyer L, R , Jabbour Zahab M, Mosna CR, Haag T, Lenormand. Not so clonal asexuals: unraveling the secret sex life of *Artemia parthenogenetica*. Evol Lett. 2021;5(2):164–174. doi:10.1002/evl3.216. 33868712 PMC8045904

[iyac123-B14] Boyer L , Jabbour-Zahab R, Joncour P, Glémin S, Haag CR, Lenormand T. Asexual Male Production by ZW Recombination in Artemia Parthenogenetica. bioRxiv0401486774; 2022. doi:10.1101/2022.04.01.486774. 36622707

[iyac123-B15] Bray NL , Pimentel H, Melsted P, Pachter L. Near-optimal probabilistic RNA-seq quantification. Nat Biotechnol. 2016;34(5):525–527. doi:10.1038/nbt.3519. 27043002

[iyac123-B16] Bushnell B. BBMap: A Fast, Accurate, Splice-Aware Aligner. No. LBNL-7065E. Berkeley (CA): Ernest Orlando Lawrence Berkeley National Laboratory, 2014.

[iyac123-B17] Chakraborty M , Baldwin-Brown JG, Long AD, Emerson JJ. Contiguous and accurate de novo assembly of metazoan genomes with modest long read coverage. Nucleic Acids Res. 2016;44(19):e147. doi:10.1093/nar/gkw654. 27458204 PMC5100563

[iyac123-B18] Charlesworth B. Model for evolution of Y chromosomes and dosage compensation. Proc Natl Acad Sci U S A. 1978;75(11):5618–5622. doi:10.1073/pnas.75.11.5618. 281711 PMC393018

[iyac123-B19] Charlesworth D , Charlesworth B, Marais G. Steps in the evolution of heteromorphic sex chromosomes. Heredity. 2005;95(2):118–128. doi:10.1038/sj.hdy.6800697. 15931241

[iyac123-B20] Charlesworth D. Young sex chromosomes in plants and animals. New Phytol. 2019;224(3):1095–1107. doi:10.1111/nph.16002. 31222890

[iyac123-B21] Danecek P , Auton A, Abecasis G, Albers CA, Banks E, DePristo MA, Handsaker RE, Lunter G, Marth GT, Sherry ST, et al; 1000 Genomes Project Analysis Group. The variant call format and VCFtools. Bioinformatics. 2011;27(15):2156–2158. doi:10.1093/bioinformatics/btr330. 21653522 PMC3137218

[iyac123-B22] De Vos S , Bossier P, Van Stappen G, Vercauteren I, Sorgeloos P, Vuylsteke M. A first AFLP-based genetic linkage map for brine shrimp *Artemia franciscana* and its application in mapping the sex locus. PLoS One. 2013;8(3):e57585. doi:10.1371/journal.pone.0057585. 23469207 PMC3587612

[iyac123-B23] De Vos S , Rombauts S, Coussement L, Dermauw W, Vuylsteke M, Sorgeloos P, Clegg JS, Nambu Z, Van Nieuwerburgh F, Norouzitallab P, et al The genome of the extremophile Artemia provides insight into strategies to cope with extreme environments. BMC Genomics. 2021;22(1):635. doi:10.1186/s12864-021-07937-z. 34465293 PMC8406910

[iyac123-B24] Dereeper A , Guignon V, Blanc G, Audic S, Buffet S, Chevenet F, Dufayard J-F, Guindon S, Lefort V, Lescot M, et al Phylogeny.fr: robust phylogenetic analysis for the non-specialist. Nucleic Acids Res. 2008;36(Web Server Issue):W465–W469. doi:10.1093/nar/gkn180. 18424797 PMC2447785

[iyac123-B25] Disteche CM. Dosage compensation of the sex chromosomes and autosomes. Semin Cell Dev Biol. 2016;56:9–18. doi:10.1016/j.semcdb.2016.04.013. 27112542 10.1016/j.semcdb.2016.04.013 PMC4955796

[iyac123-B26] Dobin A , Davis CA, Schlesinger F, Drenkow J, Zaleski C, Jha S, Batut P, Chaisson M, Gingeras TR. STAR: ultrafast universal RNA-seq aligner. Bioinformatics. 2013;29(1):15–21. doi:10.1093/bioinformatics/bts635. 23104886 PMC3530905

[iyac123-B27] Eads BD , Tsuchiya D, Andrews J, Lynch M, Zolan ME. The spread of a transposon insertion in Rec8 is associated with obligate asexuality in Daphnia. Proc Natl Acad Sci U S A. 2012;109(3):858–863. doi:10.1073/pnas.1119667109. 22215604 PMC3271927

[iyac123-B28] Elkrewi M , Moldovan MA, Picard MAL, Vicoso B. Schistosome W-linked genes inform temporal dynamics of sex chromosome evolution and suggest candidate for sex determination. Mol Biol Evol. 2021;38(12):5345–5358. doi:10.1093/molbev/msab178. 34146097 PMC8662593

[iyac123-B29] Ellegren H. Sex-chromosome evolution: recent progress and the influence of male and female heterogamety. Nat Rev Genet. 2011;12(3):157–166. doi:10.1038/nrg2948. 21301475

[iyac123-B30] Gammerdinger WJ , Toups MA, Vicoso B. Disagreement in F ST estimators: a case study from sex chromosomes. Mol Ecol Resour. 2020;20(6):1517–1525. doi:10.1111/1755-0998.13210. 32543001 PMC7689734

[iyac123-B31] Gilbert DG. Longest Protein, Longest Transcript or Most Expression, for Accurate Gene Reconstruction of Transcriptomes? bioRxiv 829184; 2019. doi:10.1101/829184.

[iyac123-B32] Grabherr MG , Haas BJ, Yassour M, Levin JZ, Thompson DA, Amit I, Adiconis X, Fan L, Raychowdhury R, Zeng Q, et al Trinity: reconstructing a full-length transcriptome without a genome from RNA-Seq data. Nat Biotechnol. 2011;29(7):644–652. doi:10.1038/nbt.1883. 21572440 PMC3571712

[iyac123-B33] Guan D , McCarthy SA, Wood J, Howe K, Wang Y, Durbin R. Identifying and removing haplotypic duplication in primary genome assemblies. Bioinformatics. 2020;36(9):2896–2898. doi:10.1093/bioinformatics/btaa025. 31971576 PMC7203741

[iyac123-B34] Guindon S , Dufayard J-F, Lefort V, Anisimova M, Hordijk W, Gascuel O. New algorithms and methods to estimate maximum-likelihood phylogenies: assessing the performance of PhyML 3.0. Syst Biol. 2010;59(3):307–321. doi:10.1093/sysbio/syq010. 20525638

[iyac123-B35] Haag CR , Theodosiou L, Zahab R, Lenormand T. Low recombination rates in sexual species and sex–asex transitions. Phil Trans R Soc B. 2017;372(1736):20160461. doi:10.1098/rstb.2016.0461. 29109224 PMC5698623

[iyac123-B36] Han X , Ren Y, Ouyang X, Zhang B, Sui L. Construction of a high-density genetic linkage map and QTL mapping for sex and growth traits in *Artemia franciscana*. Aquaculture. 2021;540:736692. doi:10.1016/j.aquaculture.2021.736692.

[iyac123-B37] Handley L-JL , Ceplitis H, Ellegren H. Evolutionary strata on the chicken Z chromosome: implications for sex chromosome evolution. Genetics. 2004;167(1):367–376. 15166161 10.1534/genetics.167.1.367 PMC1470863

[iyac123-B38] Huylmans AK , Macon A, Vicoso B. Global dosage compensation is ubiquitous in Lepidoptera, but counteracted by the masculinization of the Z chromosome. Mol Biol Evol. 2017;34(10):2637–2649. doi:10.1093/molbev/msx190. 28957502 PMC5850747

[iyac123-B39] Huylmans AK , Toups MA, Macon A, Gammerdinger WJ, Vicoso B. Sex-biased gene expression and dosage compensation on the *Artemia franciscana* Z-chromosome. Genome Biol Evol. 2019;11(4):1033–1044. doi:10.1093/gbe/evz053. 30865260 PMC6456005

[iyac123-B40] Huylmans AK , Macon A, Hontoria F, Vicoso B. Transitions to asexuality and evolution of gene expression in Artemia brine shrimp. Proc Biol Sci. 2021;288(1959):20211720. doi:10.1098/rspb.2021.1720. 34547909 PMC8456138

[iyac123-B41] Jaquiéry J , Stoeckel S, Larose C, Nouhaud P, Rispe C, Mieuzet L, Bonhomme J, Mahéo F, Legeai F, Gauthier J-P, et al Genetic control of contagious asexuality in the pea aphid. PLoS Genet. 2014;10(12):e1004838. doi:10.1371/journal.pgen.1004838. 25473828 PMC4256089

[iyac123-B42] Jo E , Lee SJ, Choi E, Kim J, Lee SG, Lee JH, Kim J-H, Park H. Whole genome survey and microsatellite motif identification of *Artemia franciscana*. Biosci Rep. 2021;41:BSR20203868. doi:10.1042/BSR20203868. 33629105 PMC7955100

[iyac123-B43] Jo E , Lee S-J, Choi E, Kim J, Lee J-H, Park H. Sex-biased gene expression and isoform profile of Brine Shrimp *Artemia franciscana* by transcriptome analysis. Anim Open Access J. MDPI. 2021;11(9):2630. doi:10.3390/ani11092630. PMC8465105 34573596

[iyac123-B44] Kampfraath AA , Dudink TP, Kraaijeveld K, Ellers J, Zizzari ZV. Male sexual trait decay in two asexual Springtail populations follows neutral mutation accumulation theory. Evol Biol. 2020;47(4):285–292. doi:10.1007/s11692-020-09511-z.

[iyac123-B45] Katoh K , Misawa K, Kuma K, Miyata T. MAFFT: a novel method for rapid multiple sequence alignment based on fast Fourier transform. Nucleic Acids Res. 2002;30(14):3059–3066. doi:10.1093/nar/gkf436. 12136088 PMC135756

[iyac123-B46] Katsuma S , Shoji K, Sugano Y, Suzuki Y, Kiuchi T. Masc-induced dosage compensation in silkworm cultured cells. FEBS Open Bio. 2019;9(9):1573–1579. doi:10.1002/2211-5463.12698. PMC6722886 31294930

[iyac123-B47] Kent WJ. BLAT–the BLAST-like alignment tool. Genome Res. 2002;12(4):656–664. doi:10.1101/gr.229202. 11932250 PMC187518

[iyac123-B48] Kiuchi T , Koga H, Kawamoto M, Shoji K, Sakai H, Arai Y, Ishihara G, Kawaoka S, Sugano S, Shimada T, et al A single female-specific piRNA is the primary determiner of sex in the silkworm. Nature. 2014;509(7502):633–636. doi:10.1038/nature13315. 24828047

[iyac123-B49] Kofler R , Pandey RV, Schlötterer C. PoPoolation2: identifying differentiation between populations using sequencing of pooled DNA samples (Pool-Seq). Bioinformatics. 2011;27(24):3435–3436. doi:10.1093/bioinformatics/btr589. 22025480 PMC3232374

[iyac123-B50] Kolmogorov M , Yuan J, Lin Y, Pevzner PA. Assembly of long, error-prone reads using repeat graphs. Nat Biotechnol. 2019;37(5):540–546. doi:10.1038/s41587-019-0072-8. 30936562

[iyac123-B51] Lahn BT , Page DC. Four evolutionary strata on the human X chromosome. Science. 1999;286(5441):964–967. doi:10.1126/science.286.5441.964. 10542153

[iyac123-B52] Langmead B , Salzberg SL. Fast gapped-read alignment with Bowtie 2. Nat Methods. 2012;9(4):357–359. doi:10.1038/nmeth.1923. 22388286 PMC3322381

[iyac123-B53] Lenormand T , Fyon F, Sun E, Roze D. Sex chromosome degeneration by regulatory evolution. Curr Biol. 2020;30(15):3001–3006.e5. doi:10.1016/j.cub.2020.05.052. 32559446

[iyac123-B54] Lenormand T , Roze D. Y recombination arrest and degeneration in the absence of sexual dimorphism. Science. 2022;375(6581):663–666. doi:10.1126/science.abj1813. 35143289

[iyac123-B55] Letunic I , Bork P. Interactive Tree Of Life (iTOL) v4: recent updates and new developments. Nucleic Acids Res. 2019;47(W1):W256–W259. doi:10.1093/nar/gkz239. 30931475 PMC6602468

[iyac123-B56] Li H , Durbin R. Fast and accurate short read alignment with Burrows-Wheeler transform. Bioinformatics. 2009;25(14):1754–1760. doi:10.1093/bioinformatics/btp324. 19451168 PMC2705234

[iyac123-B57] Li H , Handsaker B, Wysoker A, Fennell T, Ruan J, Homer N, Marth G, Abecasis G, Durbin R; 1000 Genome Project Data Processing Subgroup. The Sequence Alignment/Map format and SAMtools. Bioinformatics. 2009;25(16):2078–2079. doi:10.1093/bioinformatics/btp352. 19505943 PMC2723002

[iyac123-B58] Li H. A statistical framework for SNP calling, mutation discovery, association mapping and population genetical parameter estimation from sequencing data. Bioinformatics. 2011;27(21):2987–2993. doi:10.1093/bioinformatics/btr509. 21903627 PMC3198575

[iyac123-B59] Li D , Liu C-M, Luo R, Sadakane K, Lam T-W. MEGAHIT: an ultra-fast single-node solution for large and complex metagenomics assembly via succinct de Bruijn graph. Bioinformatics. 2015;31(10):1674–1676. doi:10.1093/bioinformatics/btv033. 25609793

[iyac123-B60] Li H. Minimap and miniasm: fast mapping and de novo assembly for noisy long sequences. Bioinformatics. 2016;32(14):2103–2110. doi:10.1093/bioinformatics/btw152. 27153593 PMC4937194

[iyac123-B61] Luo R , Liu B, Xie Y, Li Z, Huang W, Yuan J, He G, Chen Y, Pan Q, Liu Y, et al SOAPdenovo2: an empirically improved memory-efficient short-read de novo assembler. GigaScience. 2012;1(1):217X-1-18. doi:10.1186/2047-217X-1-18. PMC3626529 23587118

[iyac123-B62] Lynch M , Seyfert A, Eads B, Williams E. Localization of the genetic determinants of meiosis suppression in *Daphnia pulex*. Genetics. 2008;180(1):317–327. doi:10.1534/genetics.107.084657. 18689898 PMC2535684

[iyac123-B63] Maccari M , Gómez A, Hontoria F, Amat F. Functional rare males in diploid parthenogenetic Artemia. J Evol Biol. 2013; 26(9):1934–1948. doi:10.1111/jeb.12191. 23837914

[iyac123-B64] Maccari M , Amat F, Gómez A. Origin and genetic diversity of diploid parthenogenetic Artemia in Eurasia. PLoS One. 2013;8(12):e83348. doi:10.1371/journal.pone.0083348. 24376692 PMC3869768

[iyac123-B65] Maccari M , Amat F, Hontoria F, Gómez A. Laboratory generation of new parthenogenetic lineages supports contagious parthenogenesis in Artemia. PeerJ. 2014;2:e439. doi:10.7717/peerj.439. 25024909 PMC4081286

[iyac123-B66] Mank JE. Sex chromosome dosage compensation: definitely not for everyone. Trends Genet. 2013;29(12):677–683. doi:10.1016/j.tig.2013.07.005. 23953923

[iyac123-B67] Manni M , Berkeley MR, Seppey M, Simão FA, Zdobnov EM. BUSCO update: novel and streamlined workflows along with broader and deeper phylogenetic coverage for scoring of eukaryotic, prokaryotic, and viral genomes. Mol Biol Evol. 2021;38(10):4647–4654. doi:10.1093/molbev/msab199. 34320186 PMC8476166

[iyac123-B68] Meiklejohn CD , Landeen EL, Cook JM, Kingan SB, Presgraves DC. Sex chromosome-specific regulation in the Drosophila male germline but little evidence for chromosomal dosage compensation or meiotic inactivation. PLoS Biol. 2011;9(8):e1001126. doi:10.1371/journal.pbio.1001126. 21857805 PMC3156688

[iyac123-B69] Mignerot L , Avia K, Luthringer R, Lipinska AP, Peters AF, Cock JM, Coelho SM. A key role for sex chromosomes in the regulation of parthenogenesis in the brown alga Ectocarpus. PLoS Genet. 2019;15(6):e1008211. doi:10.1371/journal.pgen.1008211. 31194744 PMC6592573

[iyac123-B70] Mullon C , Wright AE, Reuter M, Pomiankowski A, Mank JE. Evolution of dosage compensation under sexual selection differs between X and Z chromosomes. Nat Commun. 2015;6(7720):7720. doi:10.1038/ncomms8720. 26212613 PMC4525201

[iyac123-B71] Neiman M , Sharbel TF, Schwander T. Genetic causes of transitions from sexual reproduction to asexuality in plants and animals. J Evol Biol. 2014;27(7):1346–1359. doi:10.1111/jeb.12357. 24666600

[iyac123-B72] Nougué O , Rode NO, Jabbour-Zahab R, Ségard A, Chevin L-M, Haag CR, Lenormand T. Automixis in Artemia: solving a century-old controversy. J Evol Biol. 2015;28(12):2337–2348. doi:10.1111/jeb.12757. 26356354

[iyac123-B73] Palmer DH , Rogers TF, Dean R, Wright AE. How to identify sex chromosomes and their turnover. Mol Ecol. 2019;28(21):4709–4724. doi:10.1111/mec.15245. 31538682 PMC6900093

[iyac123-B74] Parraguez M , Gajardo G, Beardmore JA. The New World Artemia species *A. franciscana* and *A. persimilis* are highly differentiated for chromosome size and heterochromatin content. Hereditas. 2009;146(2):93–103. doi:10.1111/j.1601-5223.2009.02109.x. 19490170

[iyac123-B75] Pennell MW , Mank JE, Peichel CL. Transitions in sex determination and sex chromosomes across vertebrate species. Mol Ecol. 2018;27(19):3950–3963. doi:10.1111/mec.14540. 29451715 PMC6095824

[iyac123-B76] Picard MAL , Cosseau C, Ferré S, Quack T, Grevelding CG, Couté Y, Vicoso B. Evolution of gene dosage on the Z-chromosome of schistosome parasites. eLife. 2018;7:e35684. doi:10.7554/eLife.35684. 30044216 PMC6089595

[iyac123-B77] Picard MAL , Vicoso B, Bertrand S, Escriva H. Diversity of modes of reproduction and sex determination systems in invertebrates, and the putative contribution of genetic conflict. Genes. 2021;12(8):1136. doi:10.3390/genes12081136. 34440310 PMC8391622

[iyac123-B78] Ponnikas S , Sigeman H, Abbott JK, Hansson B. Why do sex chromosomes stop recombining? Trends Genet. 2018;34(7):492–503. doi:10.1016/j.tig.2018.04.001. 29716744

[iyac123-B79] Robinson JT , Turner D, Durand NC, Thorvaldsdóttir H, Mesirov JP, Aiden EL. Juicebox.js provides a cloud-based visualization system for Hi-C data. Cell Syst. 2018;6(2):256–258.e1. doi:10.1016/j.cels.2018.01.001. 29428417 PMC6047755

[iyac123-B80] Rode NO , Jabbour-Zahab R, Boyer L, Flaven É, Hontoria F, et al The origin of asexual brine shrimps. Am Nat. 2022;200:E52–E76. doi:10.1086/720268. 35905400

[iyac123-B81] Rosin LF , Chen D, Chen Y, Lei EP. Dosage compensation in *Bombyx mori* is achieved by partial repression of both Z chromosomes in males. Proc Natl Acad Sci U S A. 2022;119(10):e2113374119. doi:10.1073/pnas.2113374119. 35239439 PMC8915793

[iyac123-B82] Rovatsos M , Kratochvíl L. Evolution of dosage compensation does not depend on genomic background. Mol Ecol. 2021;30(8):1836–1845. doi:10.1111/mec.15853. 33606326

[iyac123-B83] Ruan J , Li H. Fast and accurate long-read assembly with wtdbg2. Nat Methods. 2020;17(2):155–158. doi:10.1038/s41592-019-0669-3. 31819265 PMC7004874

[iyac123-B84] Sainz-Escudero L , López-Estrada EK, Rodríguez-Flores PC, García-París M. Settling taxonomic and nomenclatural problems in brine shrimps, Artemia (Crustacea: Branchiopoda: Anostraca), by integrating mitogenomics, marker discordances and nomenclature rules. PeerJ. 2021;9:e10865. doi:10.7717/peerj.10865. 33854829 PMC7955675

[iyac123-B85] Sandrock C , Vorburger C. Single-locus recessive inheritance of asexual reproduction in a parasitoid wasp. Curr Biol. 2011;21(5):433–437. doi:10.1016/j.cub.2011.01.070. 21353557

[iyac123-B86] Schwander T , Crespi BJ. Multiple direct transitions from sexual reproduction to apomictic parthenogenesis in Timema stick insects. Evolution. 2009;63(1):84–103. doi:10.1111/j.1558-5646.2008.00524.x. 18803687

[iyac123-B87] Schwentner M , Richter S, Rogers DC, Giribet G. Tetraconatan phylogeny with special focus on Malacostraca and Branchiopoda: highlighting the strength of taxon-specific matrices in phylogenomics. Proc R Soc B. 2018;285(1885):20181524. doi:10.1098/rspb.2018.1524. PMC6125901 30135168

[iyac123-B88] Sigeman H , Strandh M, Proux-Wéra E, Kutschera VE, Ponnikas S, Zhang H, Lundberg M, Soler L, Bunikis I, Tarka M, et al Avian neo-sex chromosomes reveal dynamics of recombination suppression and W degeneration. Mol Biol Evol. 2021;38(12):5275–5291. doi:10.1093/molbev/msab277. 34542640 PMC8662655

[iyac123-B89] Straub T , Becker PB. Dosage compensation: the beginning and end of generalization. Nat Rev Genet. 2007;8(1):47–57. doi:10.1038/nrg2013. 17173057

[iyac123-B90] Talavera G , Castresana J. Improvement of phylogenies after removing divergent and ambiguously aligned blocks from protein sequence alignments. Syst Biol. 2007;56(4):564–577. doi:10.1080/10635150701472164. 17654362

[iyac123-B91] Toman J , Flegr J. General environmental heterogeneity as the explanation of sexuality? Comparative study shows that ancient asexual taxa are associated with both biotically and abiotically homogeneous environments. Ecol Evol. 2018;8(2):973–991. doi:10.1002/ece3.3716. 29375771 PMC5773305

[iyac123-B92] Törönen P , Medlar A, Holm L. PANNZER2: a rapid functional annotation web server. Nucleic Acids Res. 2018;46(W1):W84–W88. doi:10.1093/nar/gky350. 29741643 PMC6031051

[iyac123-B93] Turner SD. qqman: an R package for visualizing GWAS results using Q-Q and Manhattan plots. J Open Source Softw. 2018;3(731). doi:10.21105/joss.00731.

[iyac123-B94] Van Stappen G. Zoogeography. In: Abatzopoulos ThJ, Beardmore JA, Clegg JS, Sorgeloos P, editors. Artemia: Basic and Applied Biology, Biology of Aquatic Organisms. Dordrecht (The Netherlands): Springer; 2002, p. 171–224.

[iyac123-B95] Vaser R , Sović I, Nagarajan N, Šikić M. Fast and accurate de novo genome assembly from long uncorrected reads. Genome Res. 2017;27(5):737–746. doi:10.1101/gr.214270.116. 28100585 PMC5411768

[iyac123-B96] Vibranovski MD , Lopes HF, Karr TL, Long M. Stage-specific expression profiling of Drosophila spermatogenesis suggests that meiotic sex chromosome inactivation drives genomic relocation of testis-expressed genes. PLoS Genet. 2009;5(11):e1000731. doi:10.1371/journal.pgen.1000731. 19936020 PMC2770318

[iyac123-B97] Vicoso B , Bachtrog D. Progress and prospects toward our understanding of the evolution of dosage compensation. Chromosome Res. 2009;17(5):585–602. doi:10.1007/s10577-009-9053-y. 19626444 PMC2758192

[iyac123-B98] Vicoso B , Emerson JJ, Zektser Y, Mahajan S, Bachtrog D. Comparative sex chromosome genomics in snakes: differentiation, evolutionary strata, and lack of global dosage compensation. PLoS Biol. 2013;11(8):e1001643. doi:10.1371/journal.pbio.1001643. 24015111 PMC3754893

[iyac123-B99] Vicoso B. Molecular and evolutionary dynamics of animal sex-chromosome turnover. Nat Ecol Evol. 2019;3(12):1632–1641. doi:10.1038/s41559-019-1050-8. 31768022

[iyac123-B100] Walters JR , Hardcastle TJ. Getting a full dose? Reconsidering sex chromosome dosage compensation in the silkworm, *Bombyx mori*. Genome Biol Evol. 2011;3:491–504. doi:10.1093/gbe/evr036. 21508430 PMC3296447

[iyac123-B101] Wang M , Kong L. pblat: a multithread blat algorithm speeding up aligning sequences to genomes. BMC Bioinformatics. 2019;20(1):28. doi:10.1186/s12859-019-2597-8. 30646844 PMC6334396

[iyac123-B102] Willforss J , Chawade A, Levander F. NormalyzerDE: online tool for improved normalization of omics expression data and high-sensitivity differential expression analysis. J Proteome Res. 2019;18(2):732–740. doi:10.1021/acs.jproteome.8b00523. 30277078

[iyac123-B103] Wright AE , Dean R, Zimmer F, Mank JE. How to make a sex chromosome. Nat Commun. 2016;7(12087):1–8. doi:10.1038/ncomms12087. PMC4932193 27373494

[iyac123-B104] Yagound B , Dogantzis KA, Zayed A, Lim J, Broekhuyse P, Remnant EJ, Beekman M, Allsopp MH, Aamidor SE, Dim O, et al A single gene causes Thelytokous parthenogenesis, the defining feature of the Cape Honeybee *Apis mellifera capensis*. Curr Biol. 2020;30(12):2248–2259.e6. doi:10.1016/j.cub.2020.04.033. 32386531

[iyac123-B105] Zeileis A , Grothendieck G. Zoo: S3 infrastructure for regular and irregular time series. J Stat Softw. 2005;14:1–27. doi:10.18637/jss.v014.i06.

[iyac123-B106] Zhou Q , Zhang J, Bachtrog D, An N, Huang Q, Jarvis ED, Gilbert MTP, Zhang G. Complex evolutionary trajectories of sex chromosomes across bird taxa. Science. 2014;346(6215):1246338. doi:10.1126/science.1246338. 25504727 PMC6445272

[iyac123-B107] Zhou C , McCarthy SA, Durbin R. YaHS: yet Another Hi-C Scaffolding Tool. bioRxiv 2022.06.09495093; 2022. doi: doi:10.1101/2022.06.09.495093. PMC9848053 36525368

