# Supplementary material for: ZW sex-chromosome evolution and contagious parthenogenesis in Artemia brine shrimp
Source: Genetics. 2022 Aug 17;222(2):iyac123. doi: 10.1093/genetics/iyac123 (PMC9526061; doi:10.1093/genetics/iyac123)
Supplement: iyac123_Supplemental_Material [file iyac123_supplemental_material.pdf]

Supplementary Material

Supplementary Datasets 3

Supplementary Figures 4

Figure S1. Phylogeny of the lineages used in the study. 4

Figure S2. Pipeline of *A. sinica* male genome assembly. 5

Figure S3. Heatmap of the Hi-C contact matrix on the *A. sinica* genome assembly. 6

Figure S4. Comparison of BUSCO scores of available Artemia genome assemblies. 7

Figure S5. Coverage patterns (log2(female/male)) and male-female F_{ST} for all the *Artemia sinica* chromosomes. 8

Figure S6. Mapping of *A. franciscana* LG6 markers to the *A. sinica* chromosomes. 9

Figure S7. Putative W-derived scaffolds in the *A. sinica* female genome assembly. 10

Figure S8. *A. franciscana* male:female F_{ST} 11

Figure S9. Phylogenetic trees of candidate W-genes shared by both *A. franciscana* and *A. sinica*. 12

Figure S10. BUSCO assessment of new transcriptome assemblies. 13

Figure S11. Coverage patterns in *A. urmiana*. 14

Figure S12. Loss of heterozygosity across the genome in the rare male (compared to its *A. parthenogenetica* sister). 15

Figure S13. Crossing scheme for identifying genomic regions linked to the spread of asexuality. 16

Figure S14 (next 4 pages). Asexual:sexual genetic differentiation and *A. parthenogenetic* Aibi Lake ancestry when using individual genotypes called with the *A. sp. Kazakhstan* genome as the reference. 17

Supplementary Tables **22**

Table S1: Genomic samples generated for this study and what steps of the analysis they were used in.. **22**

Table S2: Published RNA samples used in this study and what steps of the analysis they were used in. **22**

Table S3: Statistics for the different assembly steps of the male *A. sinica* genome. **22**

Table S4: The coordinates of the different regions of the Z chromosome and the number of the putative W and Z transcripts in those regions. **22**

Table S5: Bonferroni-corrected p-values from the Wilcoxon tests comparing the expression of the autosomes and the different regions of the Z chromosome. **23**

Table S6: Statistics for the two steps of the assembly of the *A. sp. Kazakhstan* Male Genome. **23**

Table S7: Counts of asexual females, control females, and males, among the F2 progeny of the rare male / *A. sp. Kazakhstan* backcrosses. **24**

Supplementary Datasets

Dataset S1: Genome assemblies: *A. sinica* male high quality assembly, *A. sp. Kazakhstan* male draft assembly
Dataset S2: Male transcriptome assemblies for *A. sinica* and *A. franciscana*
Dataset S3: Male and female coverage for *A. sinica*, *A. sp. Kazakhstan*, *A. urmiana*, and *A. parthenogenetica* females and rare male.
Dataset S4: *Artemia sinica* Male:female F_{ST} per 1Kb window
Dataset S5: FASTA file with candidate W scaffolds
Dataset S6: Candidate W-derived transcripts and alignments
Dataset S7: Gene expression with genomic location
Dataset S8: VCF for asexual female and rare male
Dataset S9: F_{ST} between backcrossed asexual and control females (pooled analysis)
Dataset S10: VCF of backcrossed asexual and control females (individual analysis using *A. sp. Kazakhstan* as the reference), and inferred ancestry
Dataset S11: GO and DE annotations of all the *Artemia sinica* transcripts and their locations in the *Artemia sinica* male genome.

The datasets can be downloaded from: https://doi.org/10.15479/AT:ISTA:11653.

Supplementary Figures

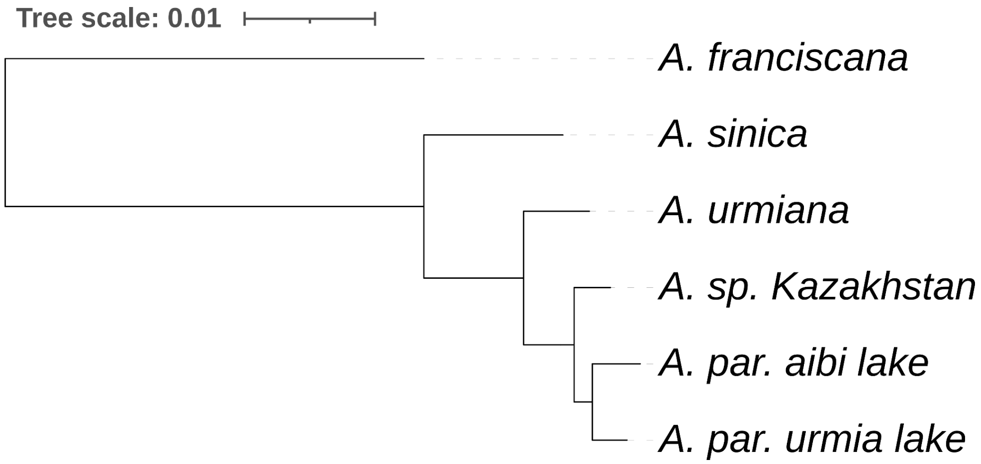

Figure S1. Phylogeny of the lineages used in the study. The phylogeny was adapted from (Huylmans et al 2021).

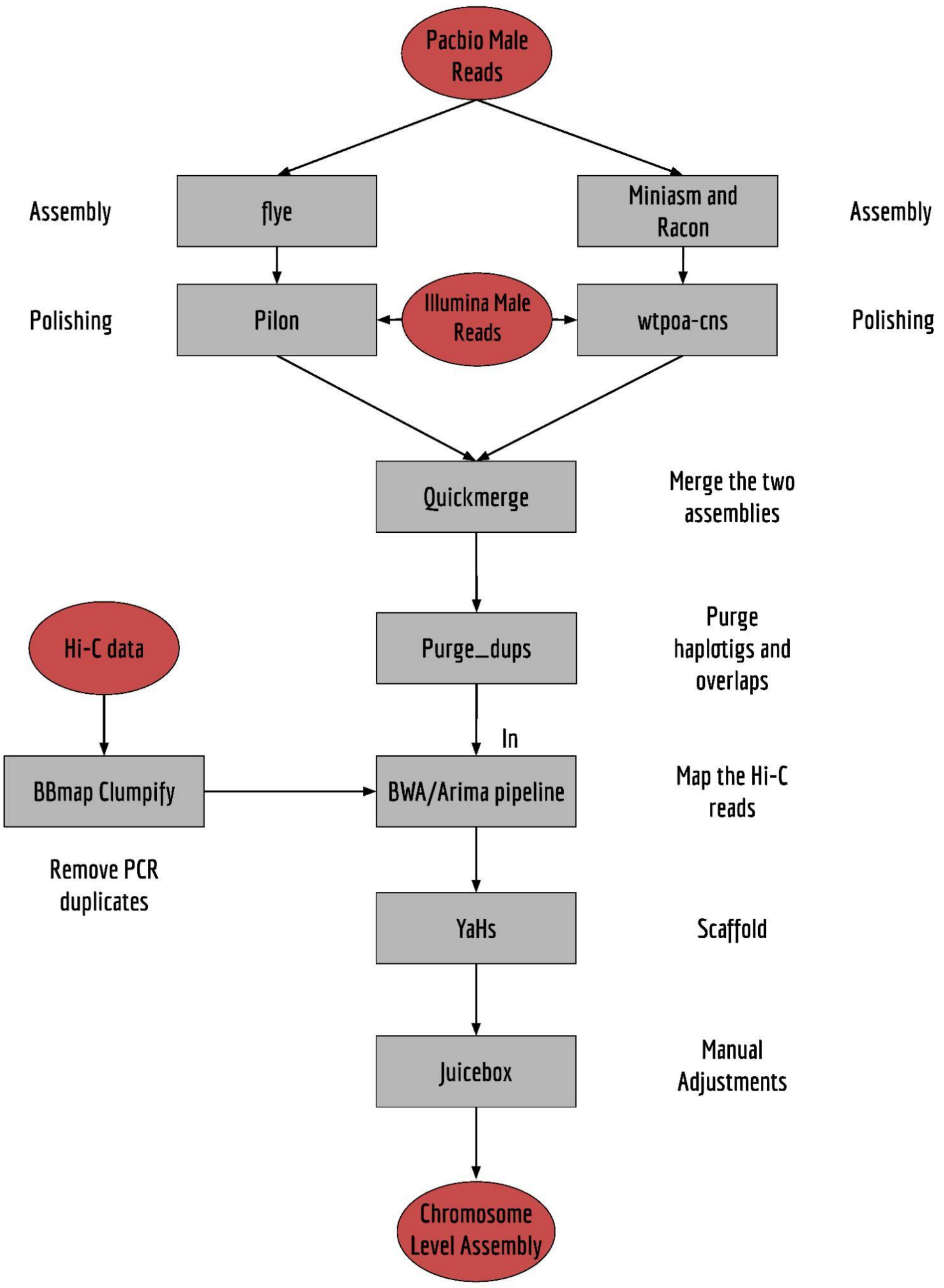

Figure S2. Pipeline of *A. sinica* male genome assembly.

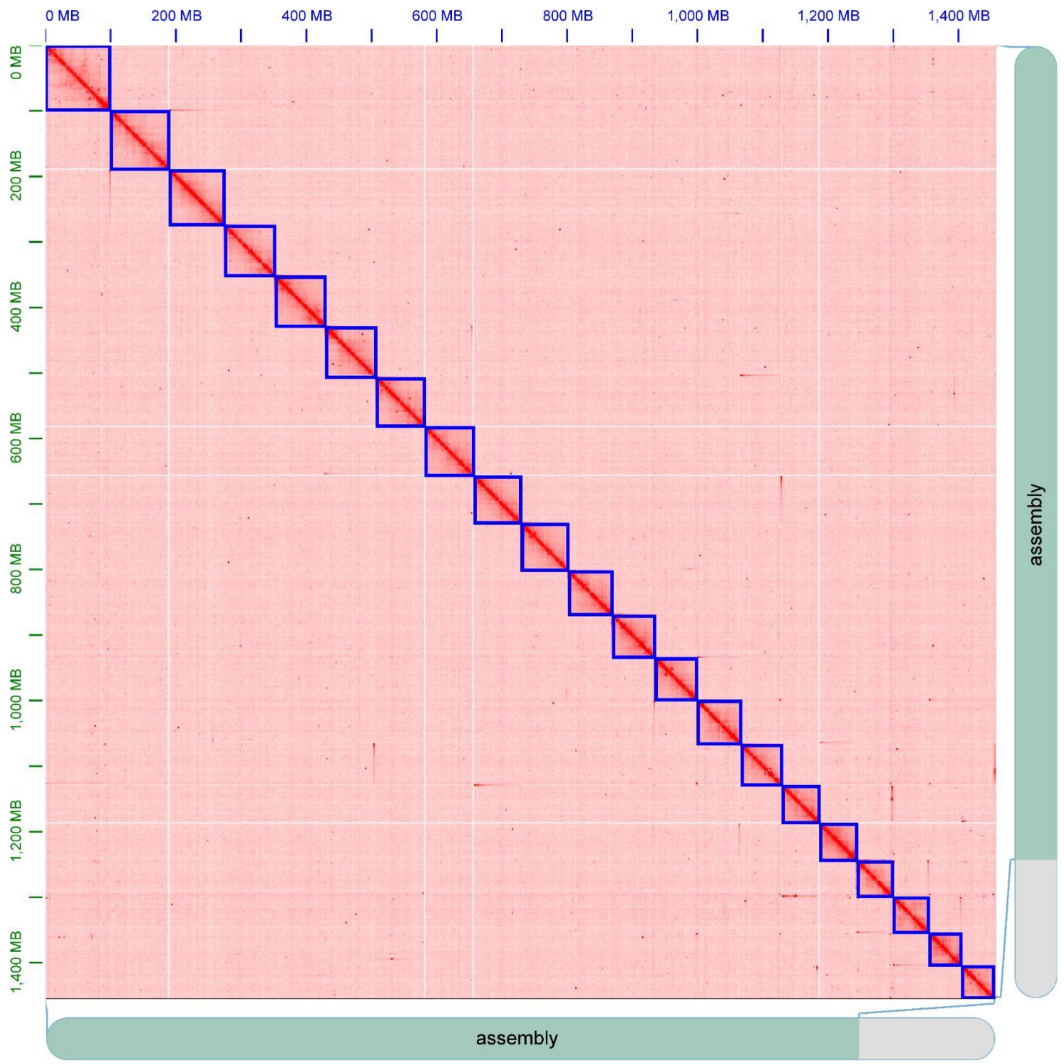

Figure S3. Heatmap of the Hi-C contact matrix on the *A. sinica* genome assembly. Blue boxes represent the largest 21 scaffolds, which correspond to the 21 chromosomes.

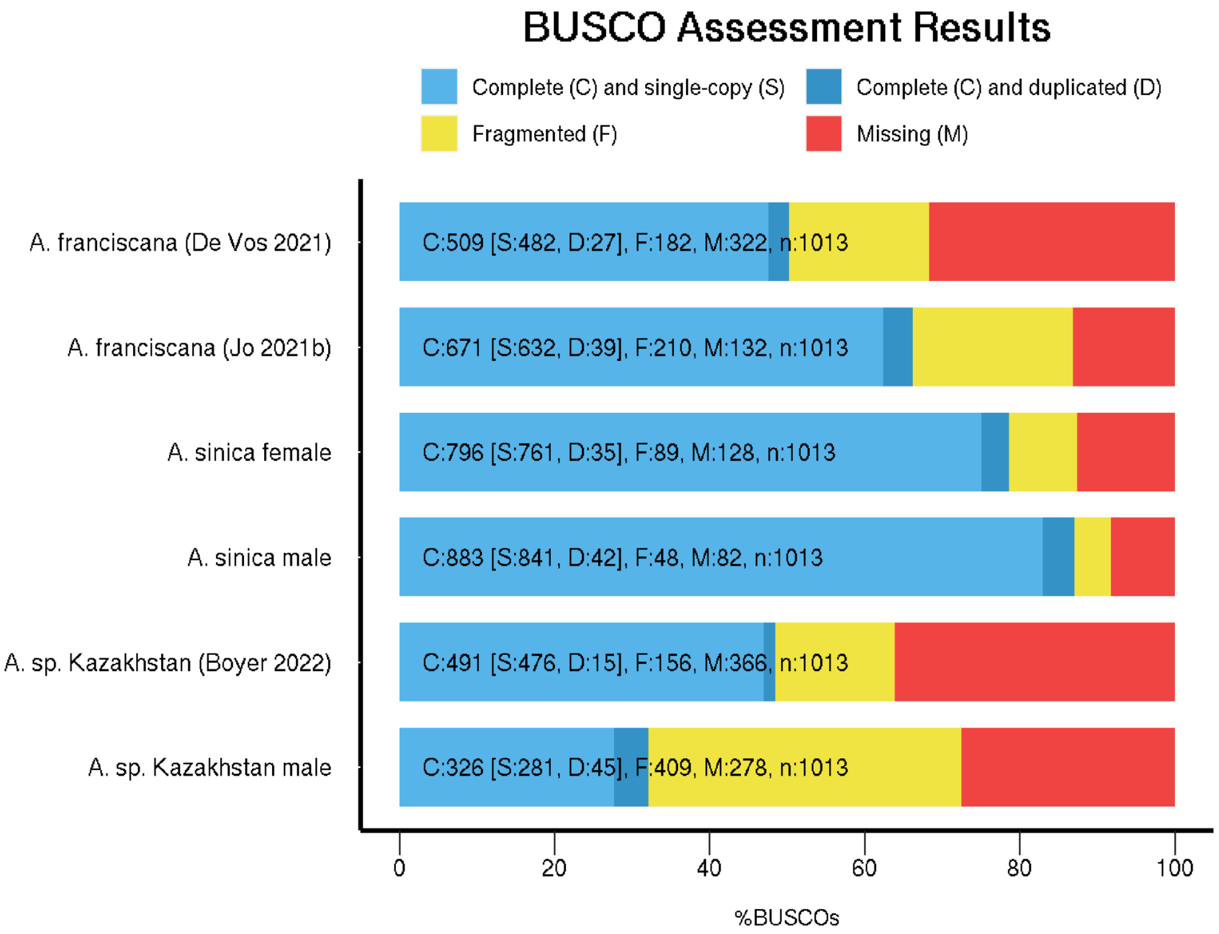

Figure S4. Comparison of BUSCO scores of available *Artemia* genome assemblies.

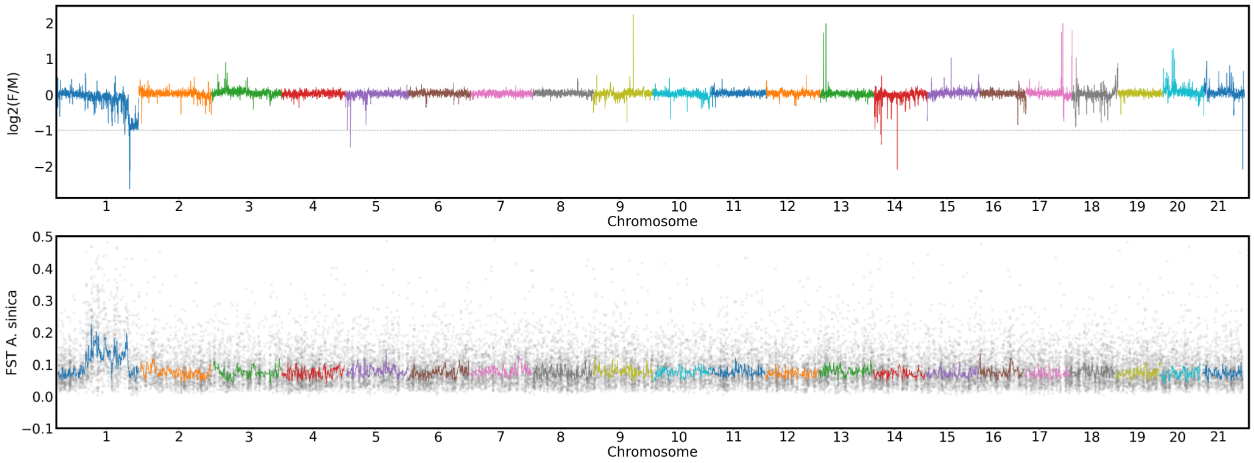

Figure S5. Coverage patterns (log2(female/male)) and male-female F_{ST} for all the *Artemia sinica* chromosomes.

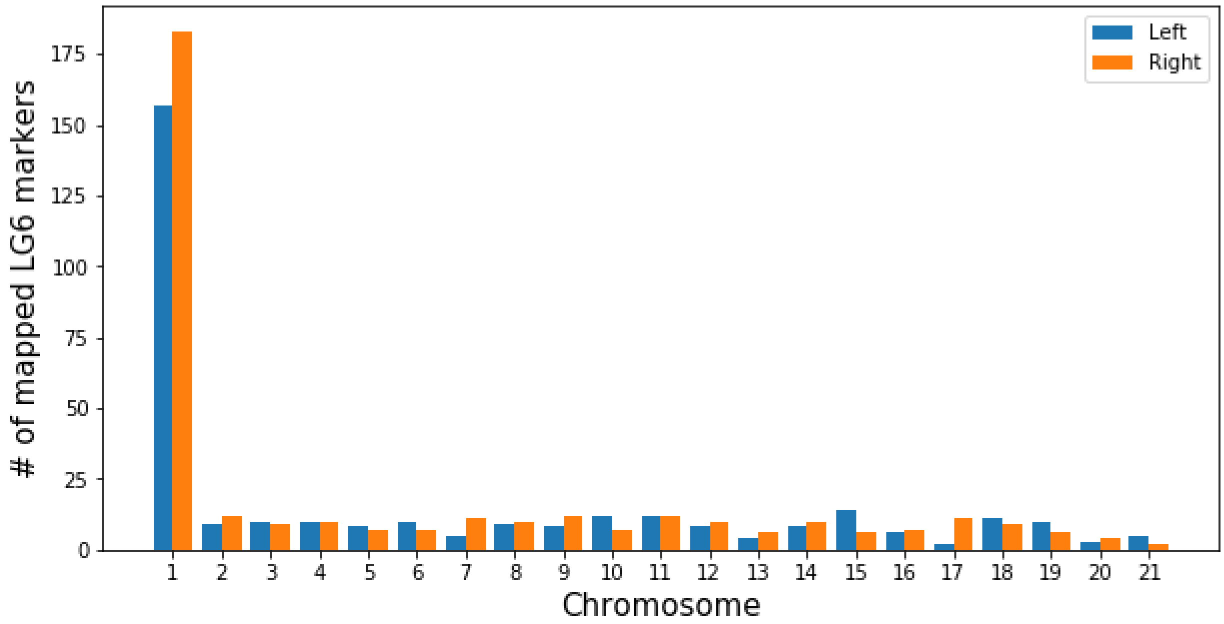

Figure S6. Mapping of *A. franciscana* LG6 markers to the *A. sinica* chromosomes. The two paired-ends of the markers were mapped individually (“Left” and “Right” markers in the plot)**.**

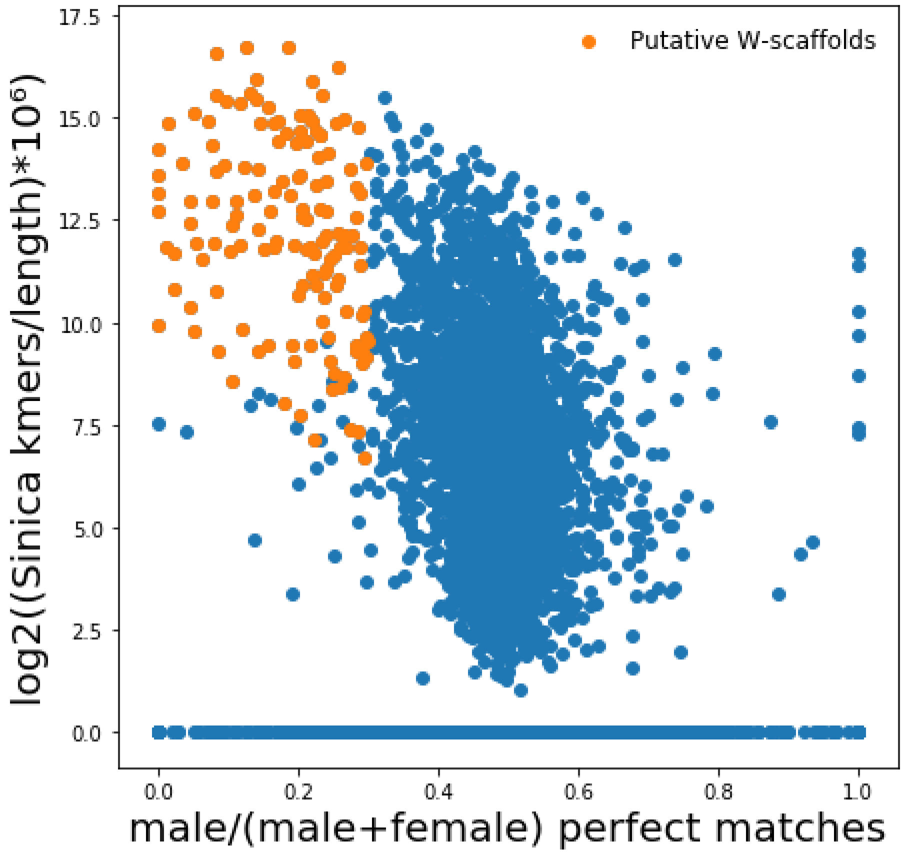

Figure S7. Putative W-derived scaffolds in the *A. sinica* female genome assembly. Scaffolds harboring more than 5 female-specific Kmers, and with more perfectly matching genomic reads in two female samples than in two male samples (male/(male+female) <=0.3), were classified as putatively W-derived (orange dots on the plot).

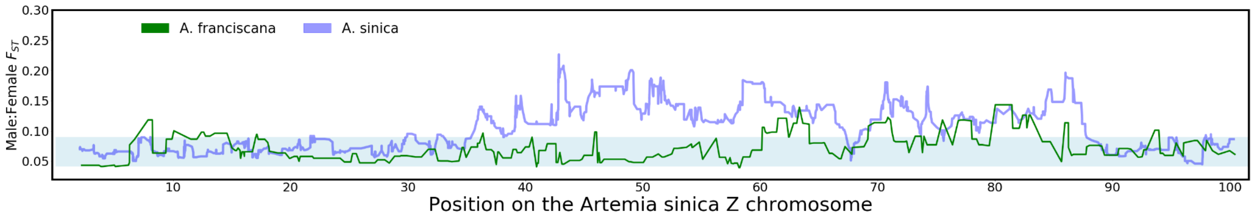

Figure S8. ***A. franciscana*** **male:female F_{ST}**

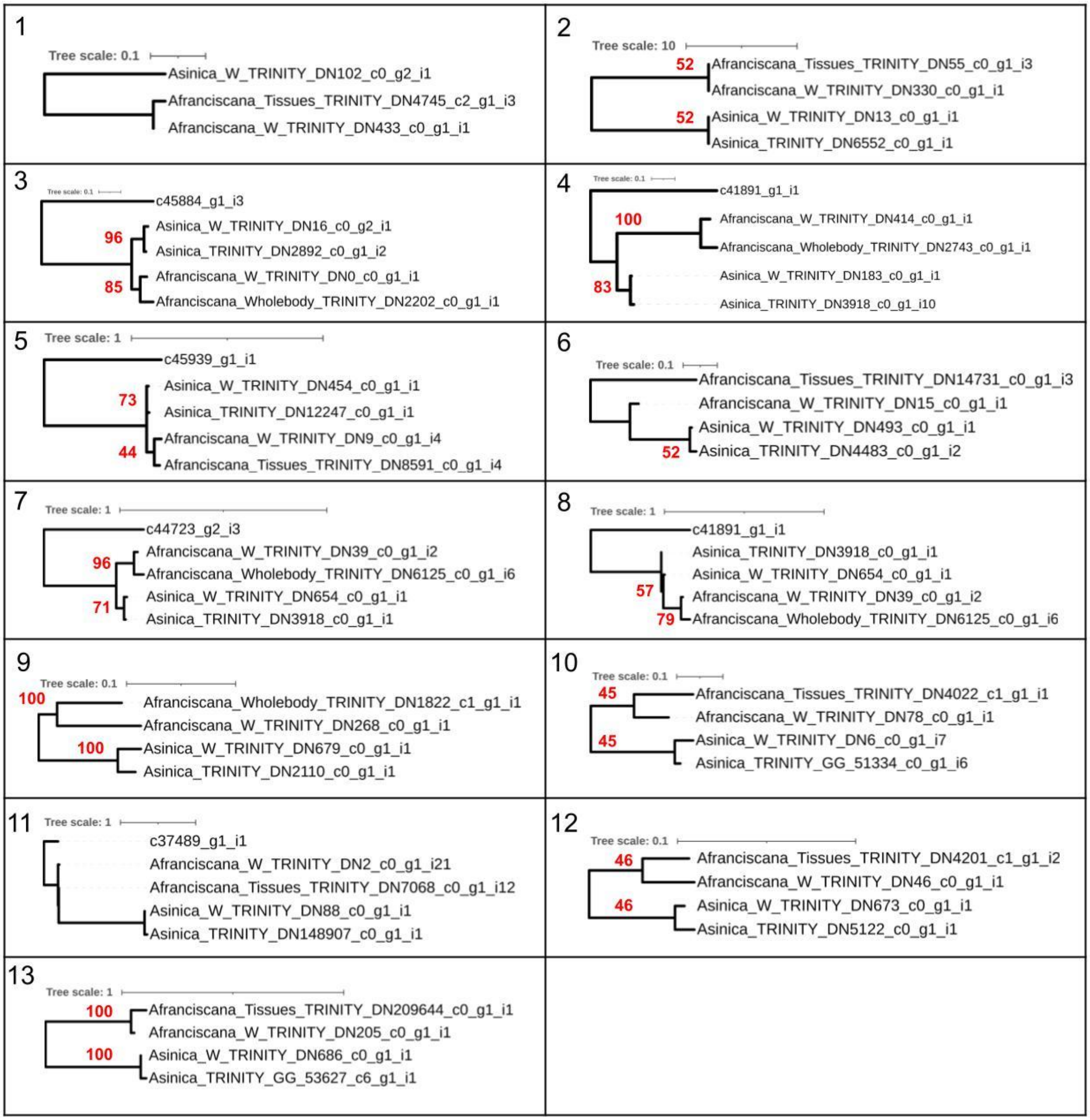

Figure S9. Phylogenetic trees of candidate W-genes shared by both *A. franciscana* and *A. sinica*. The bootstrap values are in red (based on 100 bootstraps).

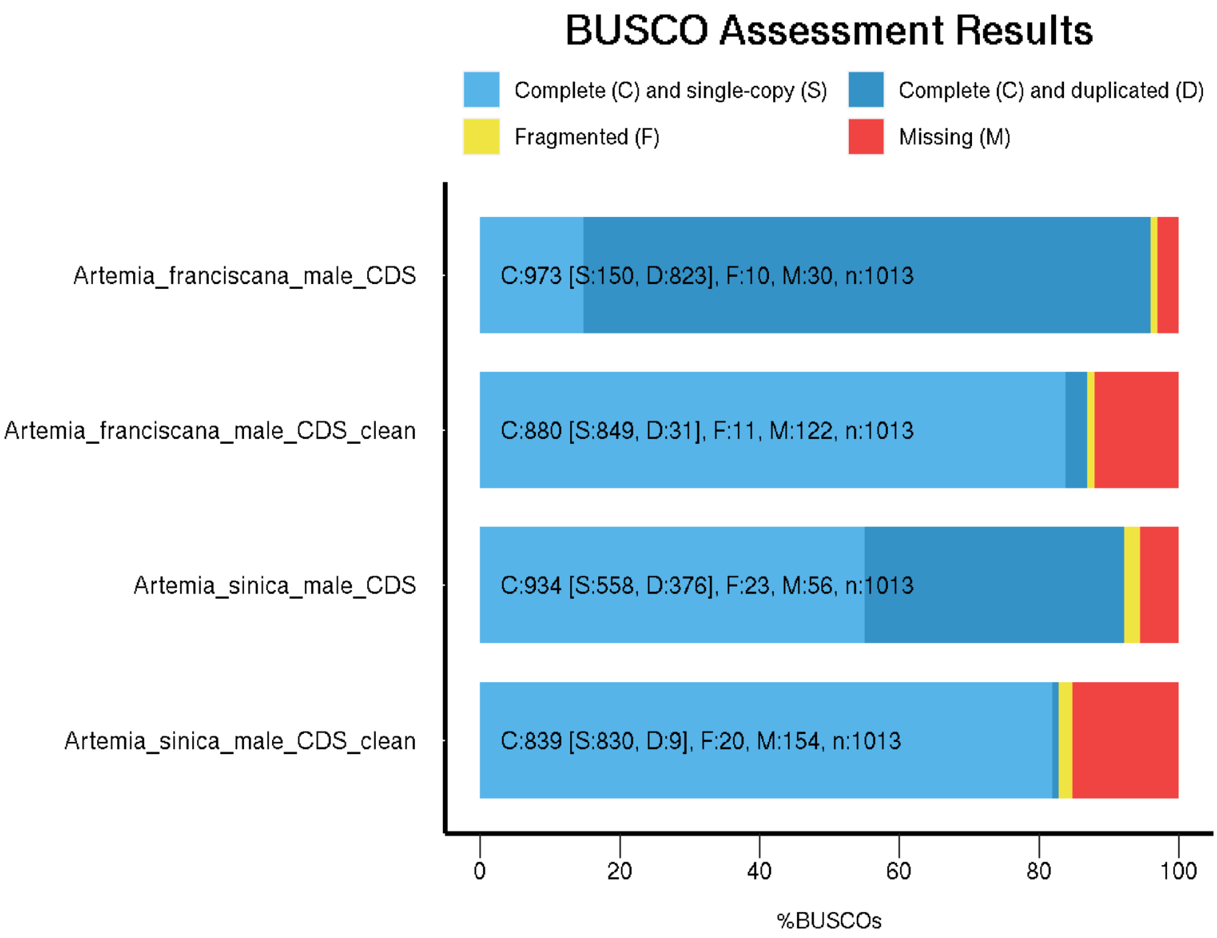

Figure S10. BUSCO assessment of new transcriptome assemblies.

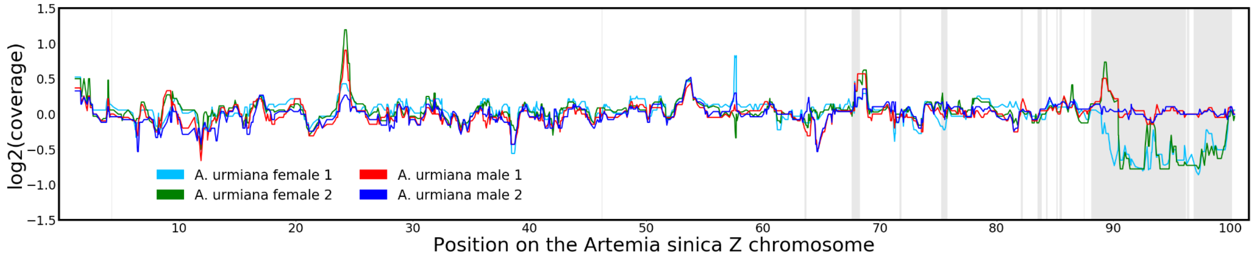

Figure S11. Coverage patterns in *A. urmiana*.

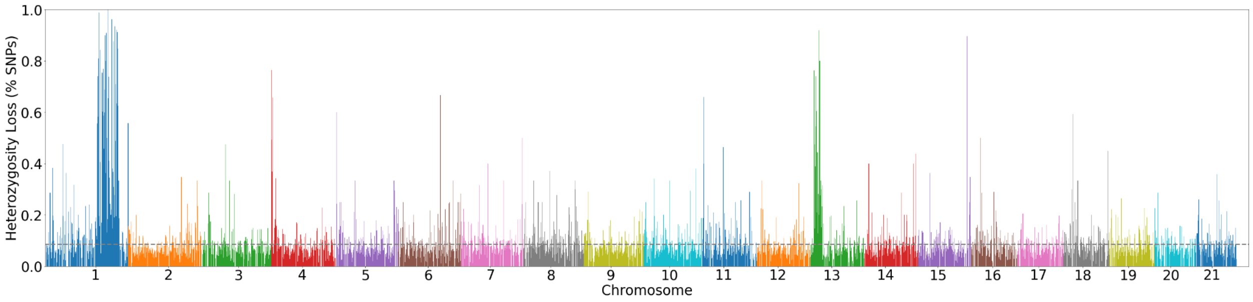

Figure S12. Loss of heterozygosity across the genome in the rare male (compared to its *A. parthenogenetica* sister).

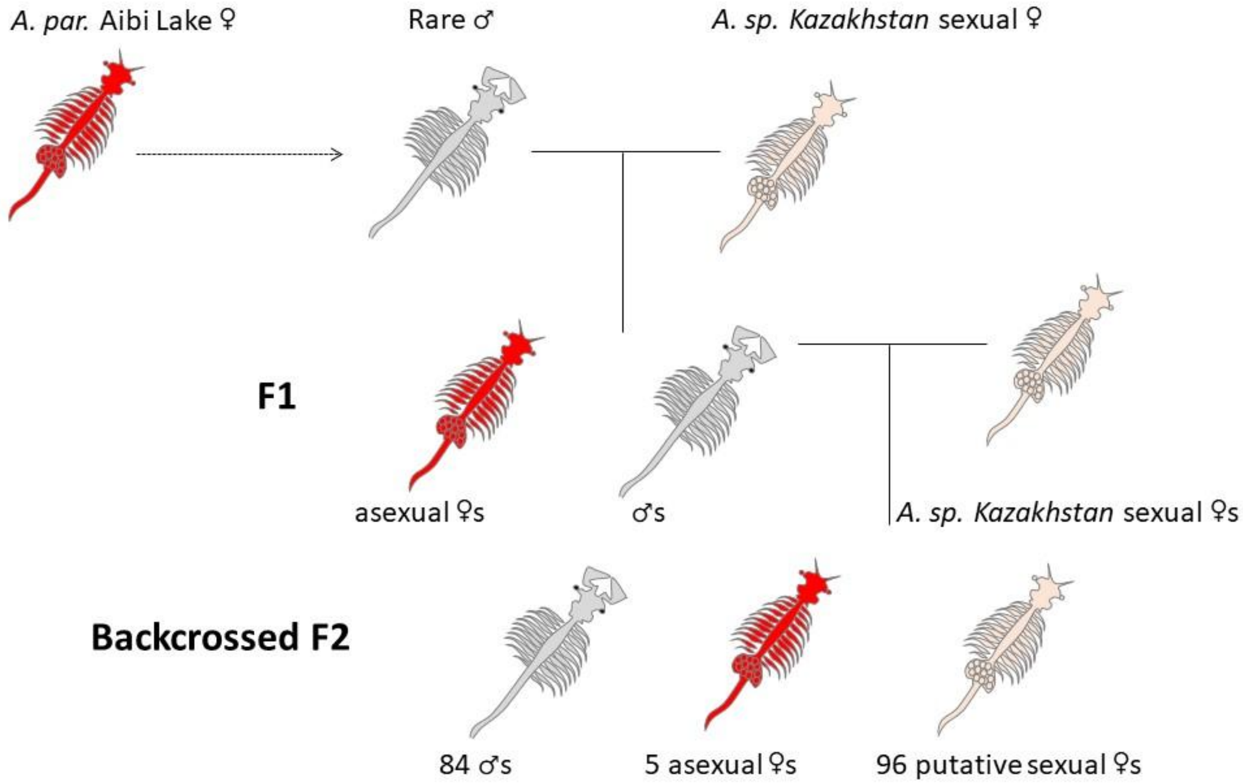

Figure S13. Crossing scheme for identifying genomic regions linked to the spread of asexuality.

Figure S14 (next 4 pages). Asexual:sexual genetic differentiation and *A. parthenogenetic Aibi Lake* ancestry when using individual genotypes called with the *A. sp. Kazakhstan* genome as the reference. For each chromosome, the top panel shows the mean F_{ST} between the 5 F2 asexual females and the 10 control putative sexual females for windows of 10KB (orange dots denote windows above the 95% percentile). The bottom panel shows the inferred ancestry of each scaffold (blue denotes *A. sp. Kazakhstan* ancestry only, red denotes both *A. sp. Kazakhstan* and *A. parthenogenetica Aibi Lake* ancestry), for each of the 5 F2 asexual females ("Asex." in the figure) and the 10 control putative sexual females ("Sex." in the figure). Orange triangles denote regions consistent with an asexuality locus, i.e. scaffolds for which all asexuals but not all putative sexuals have *Aibi Lake* ancestry. All plots were made using only SNPs that are fixed between *A. sp. Kazakhstan* and *A. parthenogenetica Aibi Lake*.

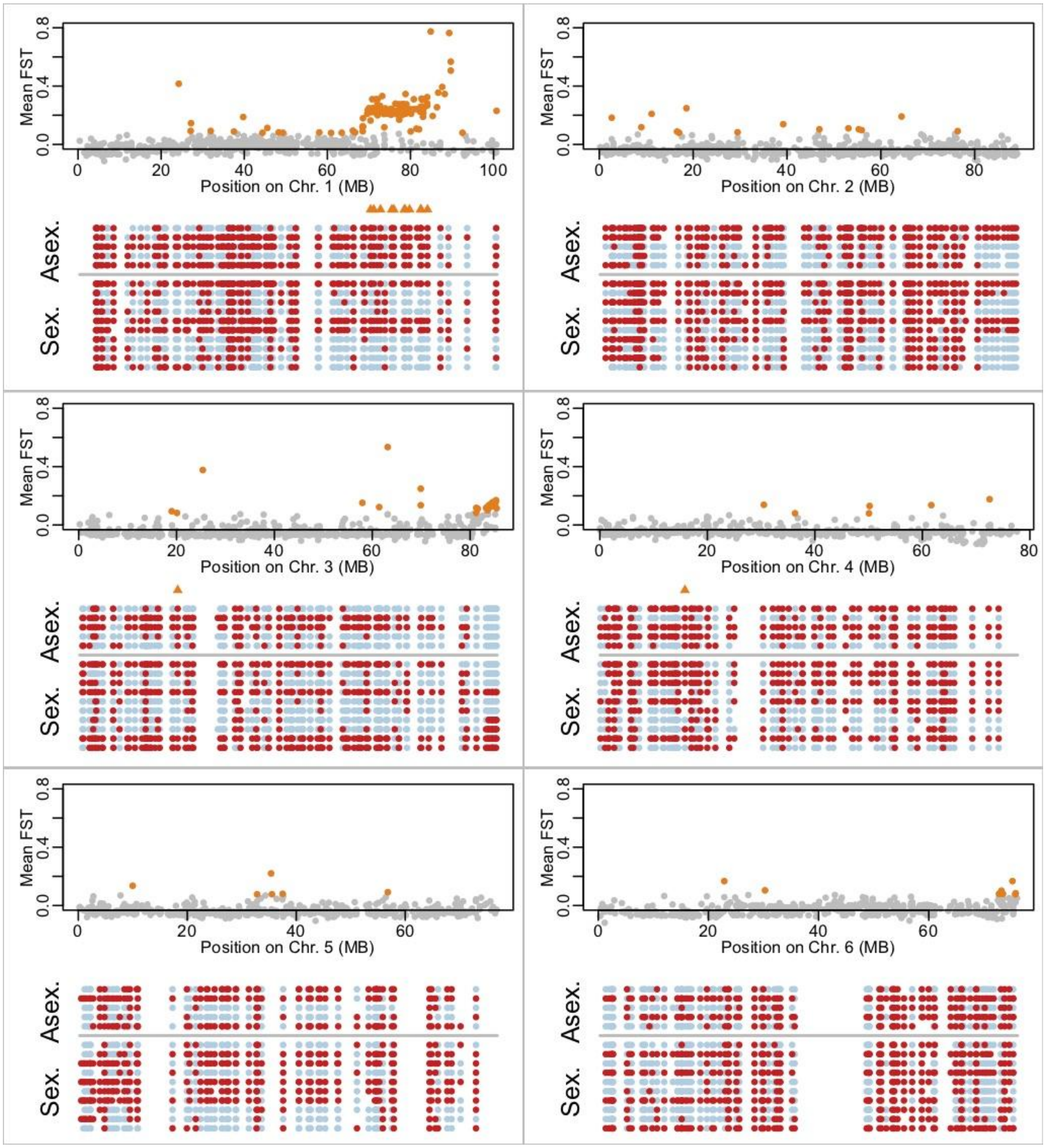
Mean FST
Position on Chr. 1 (MB)
Asex.
Sex.
Position on Chr. 2 (MB)
Position on Chr. 3 (MB)
Position on Chr. 4 (MB)
Position on Chr. 5 (MB)
Position on Chr. 6 (MB)

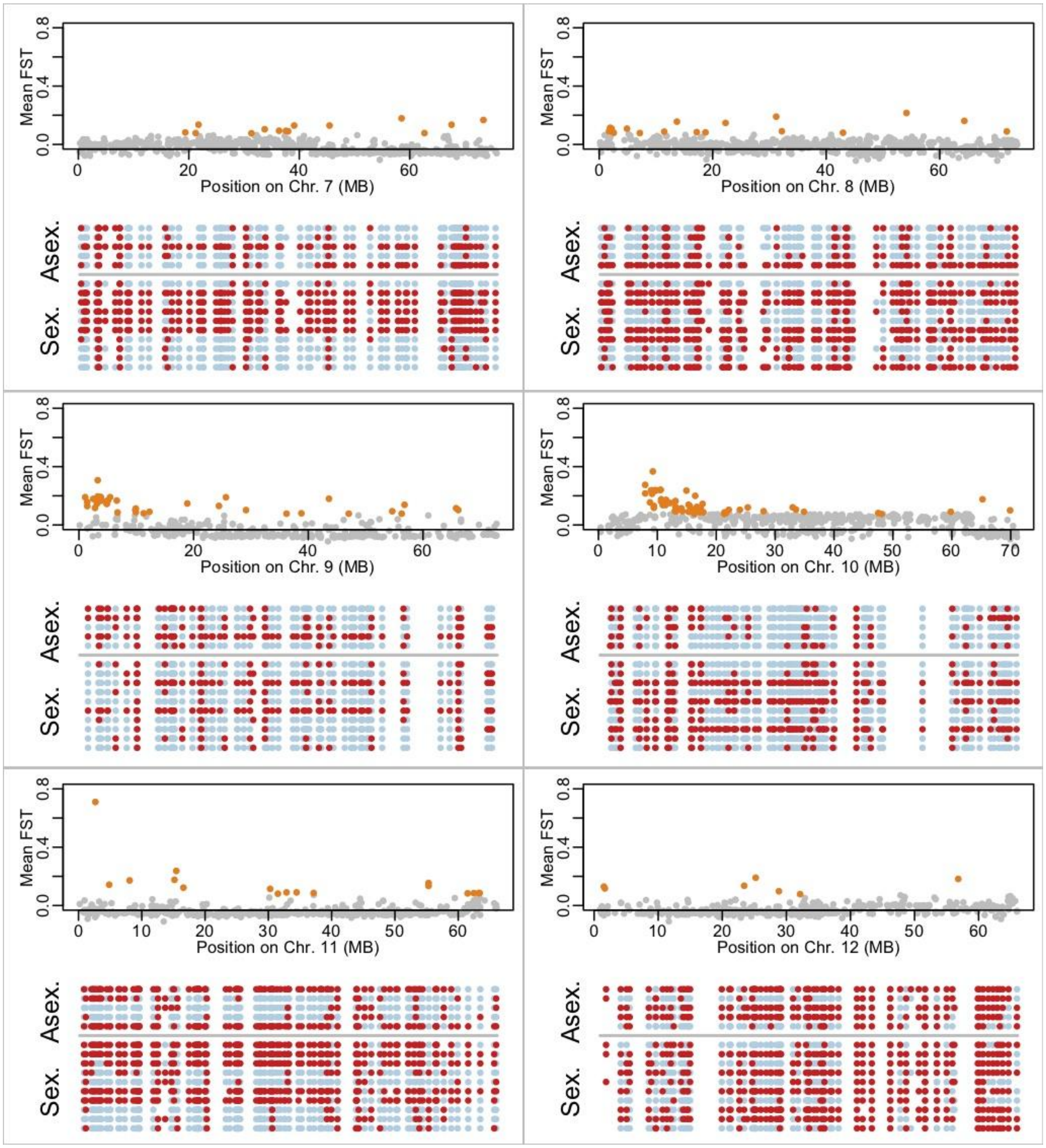
Mean FST
Position on Chr. 7 (MB)
Position on Chr. 8 (MB)
Position on Chr. 9 (MB)
Position on Chr. 10 (MB)
Position on Chr. 11 (MB)
Position on Chr. 12 (MB)
Asex.
Sex.

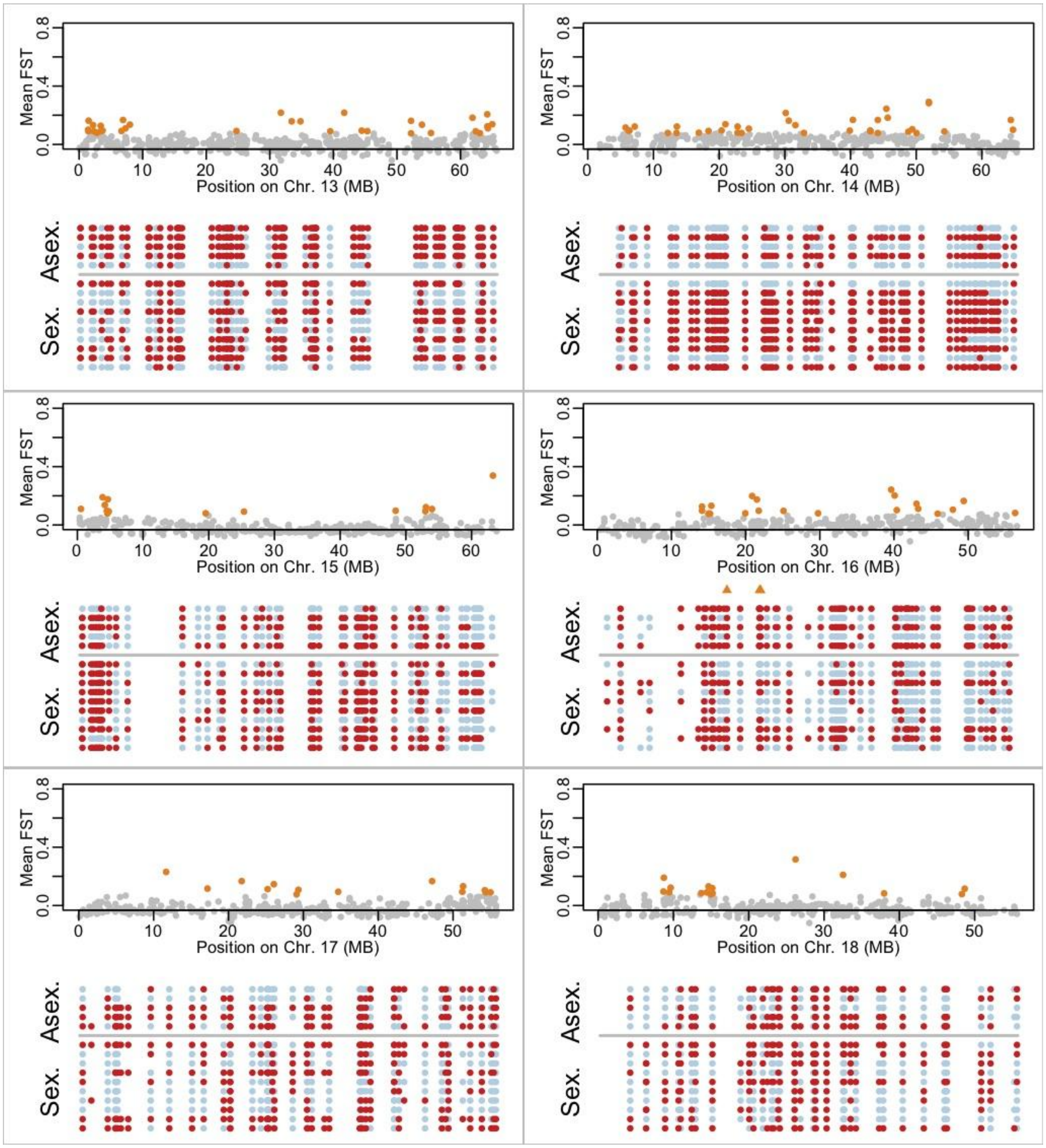
Mean FST
Position on Chr. 13 (MB)
Position on Chr. 14 (MB)
Position on Chr. 15 (MB)
Position on Chr. 16 (MB)
Position on Chr. 17 (MB)
Position on Chr. 18 (MB)
Asex.
Sex.

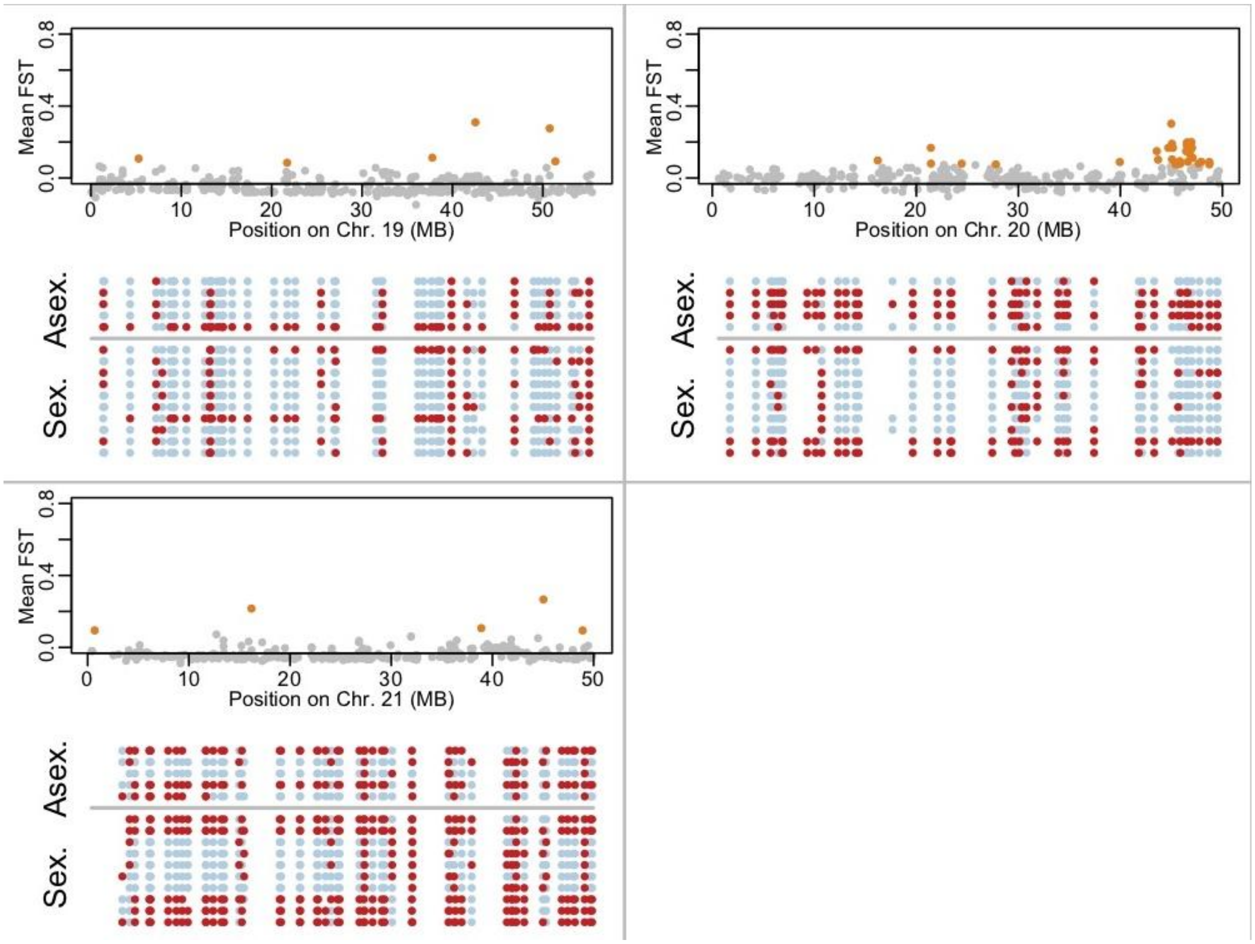
Mean FST
0.0
0.4
0.8
0
10
20
30
40
50
Position on Chr. 19 (MB)
Asex.
Sex.
Position on Chr. 20 (MB)
Position on Chr. 21 (MB)

Supplementary Tables

Table S1: Genomic samples generated for this study and what steps of the analysis they were used in. Provided as a separate excel file.

Table S2: Published RNA samples used in this study and what steps of the analysis they were used in. Provided as a separate excel file.

Table S3: Statistics for the different assembly steps of the male *A. sinica* genome.

	**Flye (+Pilon)**	**Miniasm (Racon +wtpoa-cns)**	**Quickmerge**	**Purge-dups**	**Yahs + juicebox**
**size**	1809023064	1798524669	1870672883	1700852953	1701024053
**n**	13618	5310	2946	2027	1213
**N50**	791316	570290	1969269	2176199	67194031
**N90**	86138	141470	255797	408149	1655253
**largest**	9906702	4244182	17718575	17718575	100949155
**Average**	132840.58	338705.21	634987.40	839098.64	1402328.16
**N_count**	14543	0	9276	9176	180276
**Gaps**	160	0	104	103	1812

Table S4: The coordinates of the different regions of the Z chromosome and the number of the putative W and Z transcripts in those regions.

Region	Start Coordinate (>=)	End Coordinate (<)	*Artemia sinica* W candidates	*Artemia sinica* Z	*Artemia franciscana* W candidates	*Artemia franciscana* Z
PAR	0	35322500	9	218	8	210
S1b	35322500	63525001	29	242	8	244
S1a	63525001	88115001	117	179	144	176
S0	88115001	100585001	16	84	7	91
unclassified	100585001	100949155	9	4	1	1

Table S5: Bonferroni-corrected p-values from the Wilcoxon tests comparing the expression of the autosomes and the different regions of the Z chromosome.

	Gonads p-values	Heads p-values	Thoraces p-values
autosomes-PAR	0.90912	1	1
autosomes-S1b	0.07039	1	1
autosomes-S1a	1	0.13204	1
autosomes-S0	**0.00067**	0.19521	0.55028

Table S6: Statistics for the two steps of the assembly of the *A. sp. Kazakhstan* Male Genome.

	**Megahit**	**SOAPdenovo-fusion**
**size**	1225371478	1229083815
**n**	910198	726632
**N50**	3237	4833
**N90**	440	503
**largest**	902753	996477
**Average**	1346.27	1691.48
**N_count**	0	10408426
**Gaps**	0	183782

Table S7: Counts of asexual females, control females, and males, among the F2 progeny of the rare male / A. sp. Kazakhstan backcrosses.

Cross	total individuals	males	females	dead before sexing	Asexual females	Control females sequenced
1	22	10	7	5	1	2
2	66	21	35	10	1	2
3	17	5	5	7	1	2
4	38	18	18	2	0	0
5	41	20	16	5	0	0
6	45	10	20	15	2	4
total	229	84	101	44	5	10
